# Downregulation of miRNA-205 Expression and Biological Mechanism in Prostate Cancer Tumorigenesis and Bone Metastasis

**DOI:** 10.1155/2020/6037434

**Published:** 2020-10-29

**Authors:** Yu Sun, Sheng-Hua Li, Ji-Wen Cheng, Gang Chen, Zhi-Guang Huang, Yong-Yao Gu, Hai-Biao Yan, Mao-Lin He

**Affiliations:** ^1^Division of Spinal Surgery, The First Affiliated Hospital of Guangxi Medical University, Shuangyong Road 6, Nanning, Guangxi Zhuang Autonomous Region, China 530021; ^2^Department of Urology, The First Affiliated Hospital of Guangxi Medical University, Shuangyong Road 6, Nanning, Guangxi Zhuang Autonomous Region, China 530021; ^3^Department of Pathology, The First Affiliated Hospital of Guangxi Medical University, Shuangyong Road 6, Nanning, Guangxi Zhuang Autonomous Region, China 530021

## Abstract

**Background:**

The expression and mechanism of microRNA-205 (miRNA-205) in prostate cancer (PCa) and its bone metastasis remain controversial.

**Materials and Methods:**

The expression and discriminating capability of miRNA-205 were assessed by drawing a forest plot and a summarized receiver operating characteristic (SROC) curve, using data available from 27 miRNA-array and miRNA-sequencing datasets. The miRNA-205 target genes were acquired from online prediction tools, differentially upregulated genes in PCa, and differentially expressed genes (DEGs) after miRNA-205 transfection into PCa cell lines. Functional enrichment analysis was conducted to explore the biological mechanism of miRNA-205 targets. Immunohistochemistry (IHC) was applied to verify the protein level of the hub gene.

**Results:**

The expression of miRNA-205 in the PCa group (1,461 samples) was significantly lower than that in the noncancer group (510 samples), and the downregulation of miRNA-205 showed excellent sensitivity and specificity in differentiating between the two groups. In bone metastatic PCa, the miRNA-205 level was further reduced than in nonbone metastatic PCa, and it showed a good capability in distinguishing between the two groups. In total, 153 miRNA-205 targets were screened through the three aforementioned methods. Based on the results of functional enrichment analysis, the targets of miRNA-205 were mainly enriched during chromosome segregation and phospholipid-translocating ATPase activity and in the spindle microtubule and the p53 signaling pathway. CDK1 had the highest connectivity in the PPI network analysis and was screened as one of the hub genes. A statistically significant negative correlation between miRNA-205 and CDK1 was observed. The expression of CDK1 in PCa samples was pronouncedly upregulated in terms of both the mRNA level and the protein level when compared with noncancer samples.

**Conclusion:**

miRNA-205 may play a vital role in PCa tumorigenesis and bone metastasis by targeting CDK1.

## 1. Introduction

Prostate cancer (PCa) is one of the most prevalent malignancies, being the second most common cause of death among men [1]. The high mortality associated with PCa is closely related to distant metastasis, especially bone metastasis, which often develops in PCa [2–4]. Bone metastasis exists in almost 70% of advanced PCa patients [5, 6]. There are currently a variety of treatments for localized PCa, including endocrine therapy, radiotherapy, and surgical treatment [7–9]. However, treatments are limited for patients with advanced stages of the disease and particularly bone metastatic PCa [10]. Therefore, there is an urgent need to elucidate the molecular mechanism of PCa tumorigenesis and bone metastasis and to identify new therapeutic targets.

MicroRNAs (miRNAs) are noncoding RNAs with a length of about 22 nucleotides, which affect targets' expression posttranscriptionally [11–17]. Previous studies have shown that dysregulated miRNA-205 is associated with the tumorigenesis and metastasis of a variety of malignancies. For example, the miRNA-205 level was found to be significantly higher in pancreatic cancer patients; it promotes the proliferation of pancreatic cancer cells by targeting APC [18]. Downregulated miRNA-205 is also involved in the epithelial-mesenchymal transition in breast cancer via the HMGB1-RAGE axis [19]. miRNA-205 was found to increase the proliferation and invasion capability of ovarian cancer cells by inhibiting SMAD4 and PTEN expression [20]. In PCa, some studies have observed that miRNA-205 is reduced and is a tumor suppressor [21–24]. However, Osipov et al. detected miRNA-205 expression to be predominantly higher in PCa patients than in control patients and found that the high expression of miRNA-205 distinguished PCa cases from control cases (sensitivity = 0.667; specificity = 0.773) [25]. In addition, in a study by Stephan et al., miRNA-205 expression was not statistically different among PCa and noncancer cohorts [26]. Therefore, the expression and biological mechanism of miRNA-205 in PCa need to be further explored.

**Figure 1:**
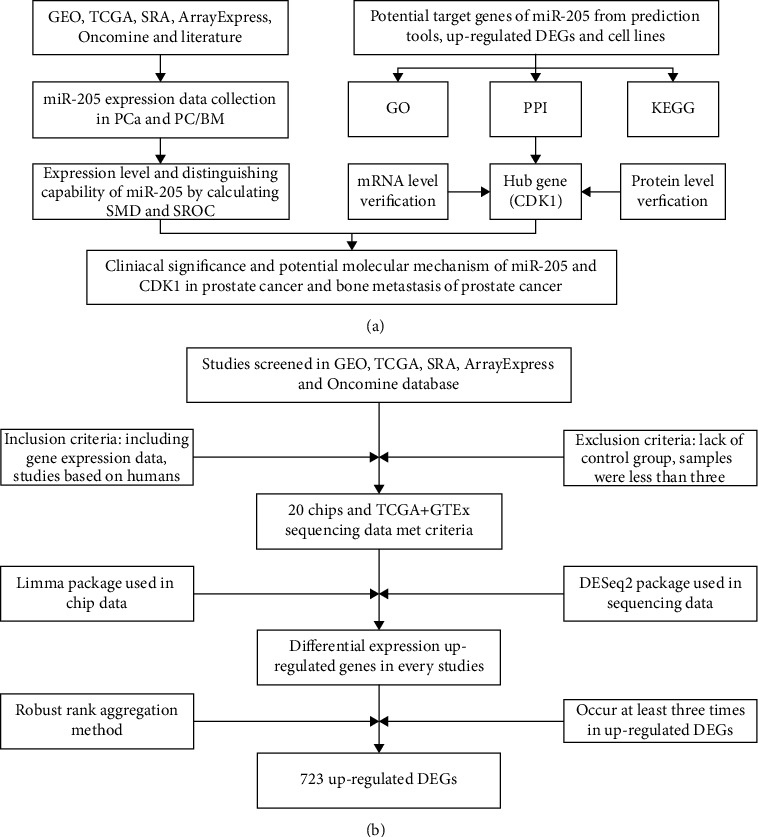
Study design. (a) Flow diagram of the study procedure. (b) Flow diagram for screening differentially upregulated genes in PCa. CDK1: cyclin-dependent kinase 1; DEG: differentially expressed gene; GEO: Gene Expression Omnibus; GO: Gene Ontology; GTEx: The Genotype-Tissue Expression; KEGG: Kyoto Encyclopedia of Genes and Genomes; PCa: prostate cancer; PPI: protein-protein interaction; SMD: standardized mean difference; SRA: Sequence Read Archive; SROC: summarized receiver operating characteristic; TCGA: The Cancer Genome Atlas.

In the present study, miRNA-array and miRNA-sequencing datasets were used to comprehensively evaluate the expression and discriminating capability of miRNA-205 in PCa and bone metastatic PCa. miRNA-205 targets were collected, and functional enrichment analysis was applied to further understand the molecular mechanism of miRNA-205 targets in PCa tumorigenesis and bone metastasis. Protein-protein interaction (PPI) network analysis screened CDK1 as one of the hub genes, and its expression in PCa was verified at the messenger RNA (mRNA) level and protein level. The design flow of the study is shown in Figure 1(a).

## 2. Materials and Methods

### 2.1. Collection of miRNA-205 Expression Data in PCa

miRNA-205 expression data in PCa were acquired from the Gene Expression Omnibus (GEO), Sequence Read Archive (SRA), Oncomine, ArrayExpress, and The Cancer Genome Atlas (TCGA) databases and related literature. The following search terms were used to search the databases: prostat ∗ AND (tumor or neoplas ∗ or malignan ∗ or adenocarcinoma or carcinoma or cancer). The studies published before December 15, 2019, were incorporated. The data inclusion criteria were as follows: (1) each study had to include a PCa group and a noncancer group, and (2) miRNA-205 expression data had to be available. The data exclusion criteria were as follows: (1) studies based on animals were excluded, and (2) studies in which fewer than three samples were used were excluded.

### 2.2. Searching for miRNA-205 Expression Data in Bone Metastatic PCa

The aforementioned databases and search formulas were applied to screen studies containing miRNA-205 expression data in bone metastatic PCa. The studies published before December 15, 2019, were incorporated. The inclusion criteria for this data were as follows: (1) each study had to include a bone metastatic PCa group and a nonbone metastatic PCa group, and (2) miRNA-205 expression data had to be available. The exclusion criteria were as follows: (1) studies based on animals were excluded, and (2) studies in which fewer than three samples were used were excluded.

### 2.3. Identification of Potential miRNA-205 Target Genes

The miRWalk2.0 archive [27], which contains 12 prediction tools, was applied to predict the targets of miRNA-205. Genes predicted by at least four tools simultaneously were considered to be targets of miRNA-205. In our study, miRNA-205 was confirmed to be downregulated in PCa. In general, miRNAs play the biological role of negatively regulating target genes. As such, we next looked for genes that were differentially upregulated in PCa as miRNA-205 target genes. The screening terms and data processing steps are shown in Figure 1(b). In order to more accurately obtain the targets of miRNA-205 in PCa, studies that interfered with miRNA-205 in PCa cell lines were included. After searching the GEO and ArrayExpress databases, only one array (GSE66498) was obtained. GSE66498 was performed in human PCa cell lines, including PC3 and DU145, and transfected with miRNA-205 and a negative control. In GSE66498, genes satisfying the standard log2FC > 1 or log2FC < −1 (*p* value < 0.05) were assumed to be the targets of miRNA-205. The overlapping of the above three units of genes was considered to represent targets of miRNA-205.

### 2.4. Gene Ontology (GO), Kyoto Encyclopedia of Genes and Genomes (KEGG), and PPI Analyses

To further understand the biological function of the miRNA-205 targets, Gene Ontology (GO) and Kyoto Encyclopedia of Genes and Genomes (KEGG) analyses were performed, employing the Database for Annotation, Visualization, and Integrated Discovery (DAVID). The PPI network analysis of miRNA-205 targets was built using the Search Tool for the Retrieval of Interacting Genes (STRING). In the PPI network, genes having the highest degree of connectivity were identified as hub genes that might play a critical role in PCa.

### 2.5. Correlation Analysis between miRNA-205 and Hub Genes

The miRNA-205 and hub gene expression data were downloaded from the GSE21032 dataset and TCGA database. The raw data were converted by log2^(*x* + 1)^. Spearman's correlation analysis was applied to assess the association between miRNA-205 and hub genes.

### 2.6. Validation of Hub Gene Expression in PCa

#### 2.6.1. mRNA Expression of Hub Genes in PCa

As shown in Figure 1(b), 21 studies containing gene expression data in PCa were incorporated. Sixteen studies containing hub gene expression data were selected to assess the mRNA expression of hub genes in PCa.

#### 2.6.2. mRNA Expression of Hub Genes in Bone Metastatic PCa

Studies involving hub gene expression data in bone metastatic PCa were screened using the aforementioned databases and search formulas. The studies published before December 15, 2019, were incorporated. The data inclusion criteria were as follows: (1) each study had to include a bone metastatic PCa group and a nonbone metastatic PCa group, and (2) hub gene expression data had to be available. The exclusion criteria were as follows: (1) studies based on animals were excluded, and (2) studies in which fewer than three samples were used were excluded.

#### 2.6.3. Protein Expression of Hub Genes in PCa by Immunostaining

IHC was used to evaluate the protein expression of hub genes in PCa samples and noncancer samples and to elucidate the relationships between hub genes and clinical characteristics of PCa. A total of 4 tissue microarrays containing 160 PCa samples and 61 noncancer samples were obtained from Superbiotek (Shanghai, China) and Fanpu (Guilin, China). The immunohistochemistry (IHC) procedure was performed according to the manufacturer's protocols. Rabbit polyclonal anti-CDK1 (1 : 50 dilution; catalog no. ab131450, Abcam, Cambridge, MA, USA) was applied for IHC. The immunoreactive score (IRS) was calculated as previously described [28].

### 2.7. Clinical Significance of miRNA-205 and Hub Genes in PCa

The clinical characteristics of age, tumor (T) stage, node (N) stage, metastasis (M) stage, Gleason score, recurrence, and survival time in PCa patients were downloaded from TCGA database. The relationships between miRNA-205, hub genes, and clinical characteristics were analyzed using Student's *t*-test.

### 2.8. Statistical Analysis

The miRNA-205 and hub gene expression data were extracted from the screened studies, and the raw data was processed by log2^(*x* + 1)^. Student's *t*-test was employed to estimate the differential expression of miRNA-205 and hub genes between the two groups using SPSS 22.0 (IBM, Chicago, IL, USA). Based on the expression of miRNA-205 and hub genes, the cutoff value for each study was calculated. A receiver operating characteristic (ROC) curve was applied to test the discriminating capability of miRNA-205 and hub genes in each study. A SROC curve was employed to estimate the overall discriminating capability of miRNA-205 and hub genes. The standardized mean difference (SMD) and 95% confidence interval (CI) were determined using the Stata 12.0 software (Stata Corp., College Station, TX, USA) to evaluate the differential expression of miRNA-205 and hub genes between two groups. The *I*^2^ statistic and chi-squared test were applied to determine the heterogeneity of the studies. When *p* > 0.05 and *I*^2^ < 50%, the studies were considered homogeneous, and a fixed-effects model was used. When *p* < 0.05 and *I*^2^ > 50%, the studies were considered heterogeneous, and a random-effects model was used. A *p* value < 0.05 was considered statistically significant.

## 3. Results

### 3.1. miRNA-205 Expression in PCa

**Figure 2:**
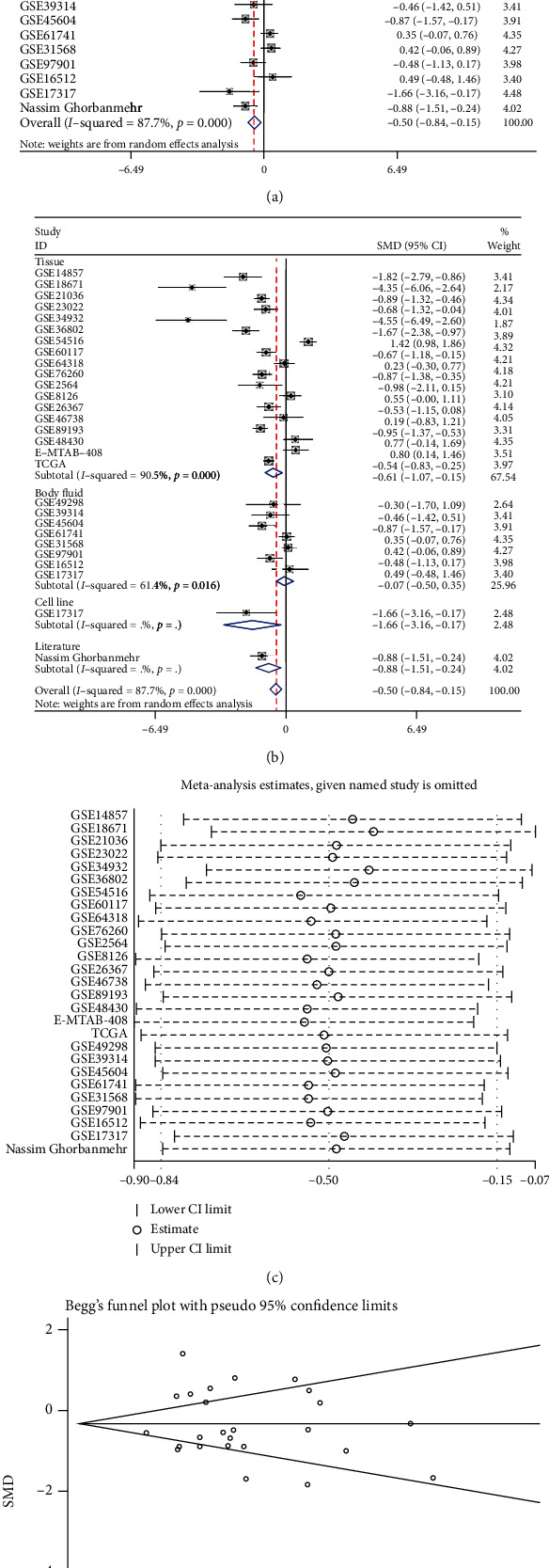
The miRNA-205 expression level in PCa. (a) Forest diagram of 27 studies assessing miRNA-205 in PCa. (b) Subgroup analysis of miRNA-205 expression based on the sample type. (c) Sensitivity analysis of miRNA-205 expression in PCa. (d) Begg's funnel diagram, which indicated no publication bias. 95% CI: 95% confidence interval; SMD: standardized mean difference.

A total of 27 eligible studies were included (Table 1). With respect to these, 12 studies showed that the expression of miRNA-205 in the PCa samples was significantly lower than that in the noncancer samples; 3 studies showed that PCa samples had a higher miRNA-205 expression compared with noncancer samples; and miRNA-205 was not significantly different between the PCa samples and the noncancer samples in 12 remaining studies (Figs. S1 and S2). The result (SMD = −0.50; 95% CI = −0.84 to −0.15; and *I*^2^ = 87.7%) indicated that miRNA-205 expression was significantly lower in the PCa group (1,461 samples) than in the noncancer group (570 samples) (Figure 2(a)).

**Figure 3:**
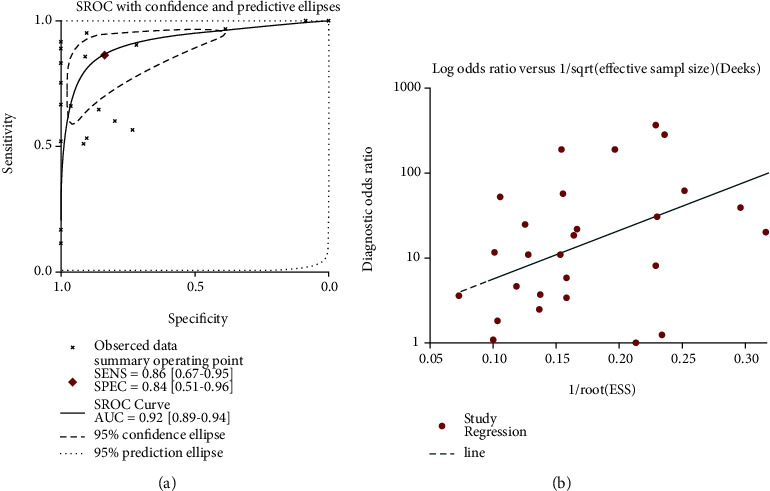
Diagnostic capability of miRNA-205 in PCa. (a) The SROC curve, which indicated that miRNA-205 had an outstanding capability in discriminating PCa from non-PCa. (b) Funnel diagram, which suggested no publication bias. PCa: prostate cancer; SROC: summarized receiver operating characteristic.

Due to the significant heterogeneity that was observed, we first performed a subgroup analysis based on the sample type, and the results showed that the sample type was not the source of the heterogeneity (Figure 2(b)). To further probe the origin of the heterogeneity, we implemented a sensitivity analysis; however, the result showed that no study caused the heterogeneity (Figure 2(c)). The results of the funnel plot indicated no publication bias (Figure 2(d)). The SROC curve showed that miRNA-205 had an excellent capacity to discriminate PCa samples from noncancer samples (area under the curve (AUC) = 0.92; sensitivity = 0.86; and specificity = 0.84) (Figure 3(a)). No publication bias was observed (Figure 3(b)). The diagnostic capacity of miRNA-205 in each study is shown in Figs. S3 and S4.

### 3.2. miRNA-205 Expression in Bone Metastatic PCa

According to the inclusion and exclusion criteria, three studies were incorporated (Table S1). The miRNA-205 expression was significantly reduced in bone metastatic samples in all three studies (Figs. S5A, S5C, and S5E). The AUC values of miRNA-205 from GSE21036, GSE26964, and TCGA were 0.901, 0.964, and 0.778, respectively (Figs. S5B, S5D, and S5E).

### 3.3. Identification of miRNA-205 Target Genes

**Figure 4:**
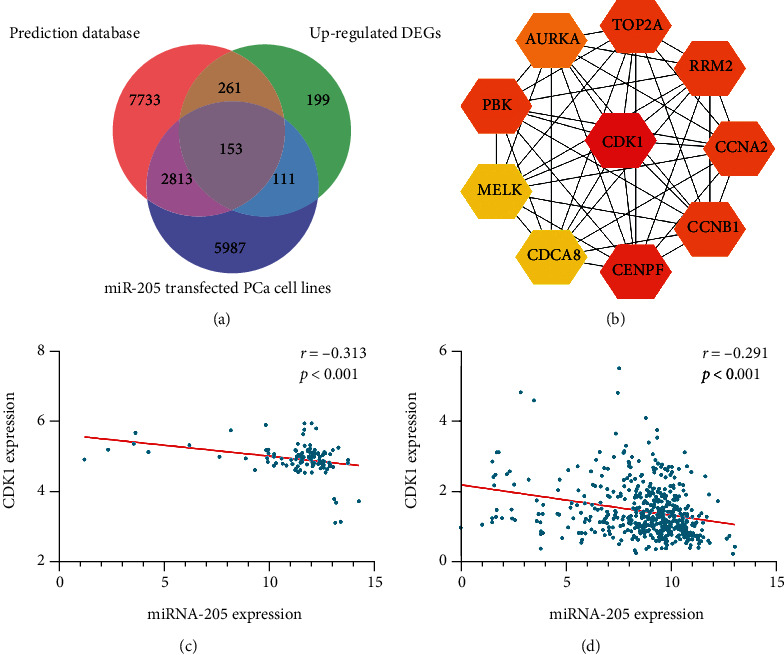
Potential target genes of miRNA-205 and hub genes screening. (a) A total of 153 genes were finally obtained as potential targets of miRNA-205. (b) The PPI networks of the target genes of miRNA-205. (c) Negative correlation between miRNA-205 and CDK1 in the GSE21032 dataset. (d) Negative correlation between miRNA-205 and CDK1 in TCGA database.

The miRNA-205 targets predicted by at least 4 tools were screened from the miRWalk2.0 database, resulting in a total of 10,959 genes. According to the screening terms and data processing procedure in Figure 1(b), a total of 21 studies were incorporated, and 723 genes were identified as targets of miRNA-205. In the GSE44698 dataset, 9,063 genes satisfying the standard log2FC < −1 (*p* value < 0.05) were screened as miRNA-205 targets. After intersecting the above 3 parts of genes, 153 genes were finally obtained as potential targets of miRNA-205 (Figure 4(a)).

### 3.4. GO, KEGG, and PPI Analyses

**Figure 5:**
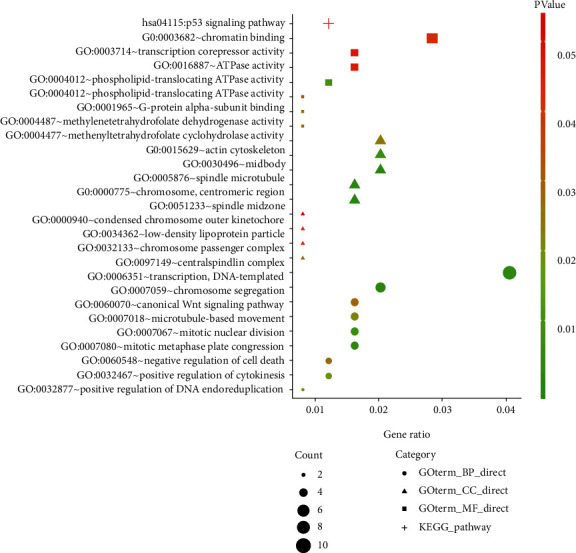
Functional and pathway enrichment analyses of the target genes of miRNA-205.

To further understand the biological function of miR-205 in PCa, GO and KEGG analyses were performed in DAVID. Among the GO analysis results, chromosome segregation; transcription, DNA-templated; and mitotic metaphase plate congression were the three most significant biological processes (BP). The results of the cellular component (CC) analysis indicated that miRNA-205 potential targets were mainly concentrated in the spindle microtubule; spindle midzone; and chromosome, centromeric region. For molecular function (MF), the three significantly involved entries were phospholipid-translocating ATPase activity, methylenetetrahydrofolate dehydrogenase activity, and methenyltetrahydrofolate cyclohydrolase activity (Table S2 and Figure 5). The KEGG results indicated that miRNA-205 targets were mainly concentrated in the p53 signaling pathway (Table S2 and Figure 5), while the PPI network analysis showed that CDK1 had the highest connectivity among the miRNA-205 targets and was screened as one of the hub genes (Figure 4(b)).

### 3.5. Correlation Analysis between miRNA-205 and CDK1

**Figure 6:**
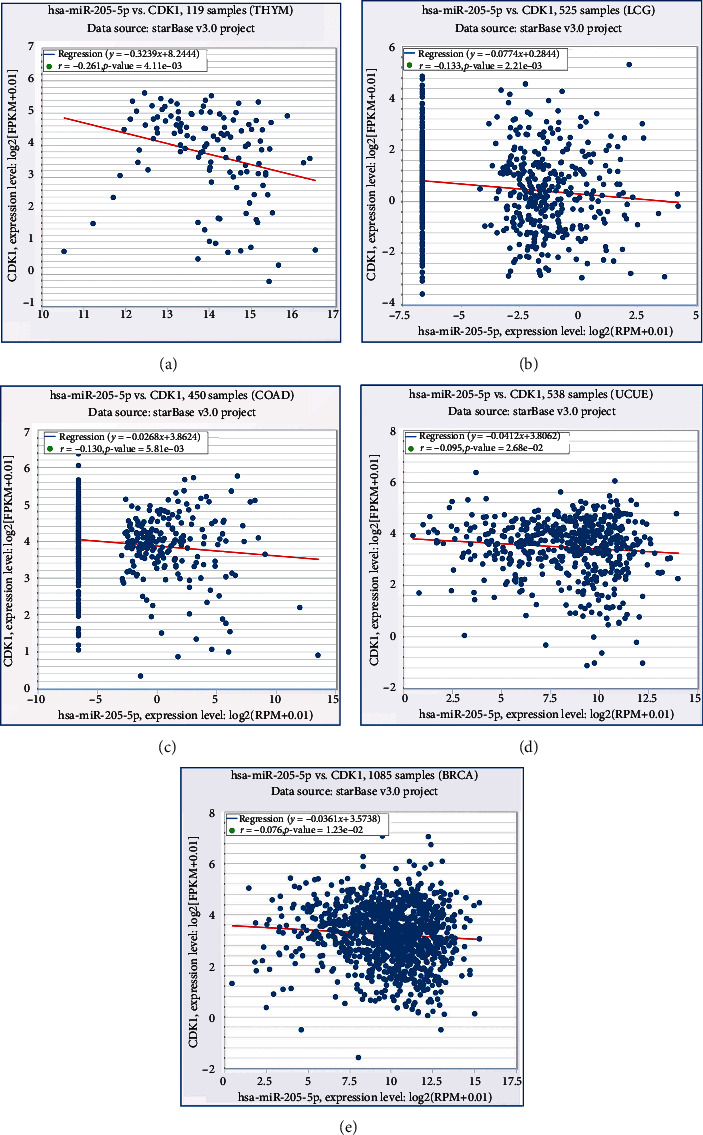
Correlations between miRNA-205 and CDK1 on the basis of the starBase v3.0 pan-cancer analysis project. (a) Thymoma. (b) Brain lower grade glioma. (c) Colon adenocarcinoma. (d) Uterine corpus endometrial carcinoma. (e) Breast invasive carcinoma.

Spearman's correlation analysis was applied to detect the association between miRNA-205 and CDK1 in PCa. The result showed that there was a significantly reverse connection between miRNA-205 and CDK1 in the GSE21032 dataset and TCGA database (Figures 4(c) and 4(d)). In addition, we acquired the expression of miRNA-205 and CDK1 in different types of cancer from starBase v3.0. A negative correlation between miRNA-205 and CDK1 was observed in thymoma, brain lower grade glioma, colon adenocarcinoma, uterine corpus endometrial carcinoma, and breast invasive carcinoma (Figure 6).

### 3.6. Validation of CDK1 Expression in PCa

#### 3.6.1. CDK1 mRNA Expression in PCa

**Figure 7:**
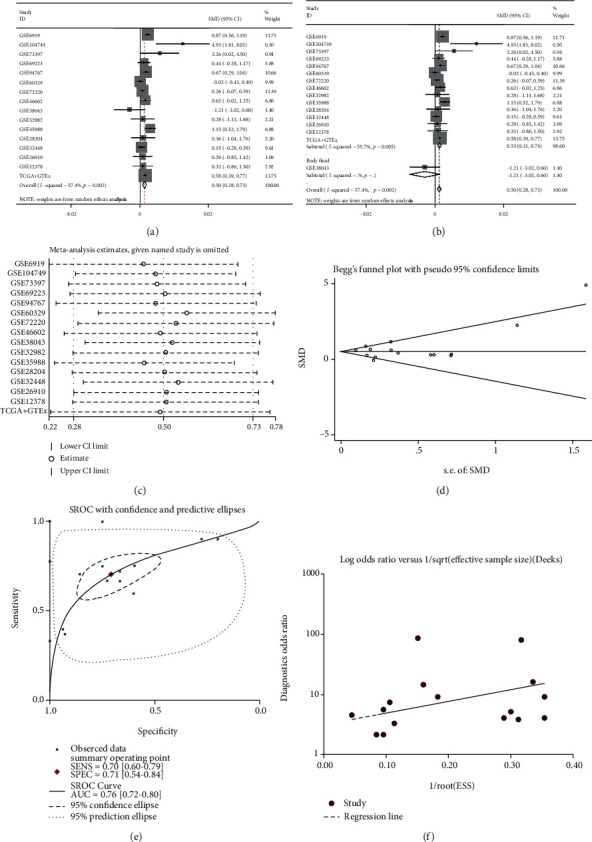
CDK1 expression level and diagnostic capability in PCa. (a) Forest diagram of CDK1 expression in PCa. (b) Subgroup analysis of CDK1 expression based on the sample type. (c) Sensitivity analysis of CDK1 expression in PCa. (d) Begg's funnel diagram, which indicated no publication bias. (e) The SROC curve, which indicated that CDK1 had an acceptable capability in discriminating PCa from non-PCa. (f) Funnel diagram, which suggested no publication bias. 95% CI: 95% confidence interval; CDK1: cyclin-dependent kinase 1; PCa: prostate cancer; SMD: standardized mean difference; SROC: summarized receiver operating characteristic.

Sixteen studies from the mRNA-array and mRNA-sequencing datasets were used to verify the mRNA expression level of CDK1 (Table 2). Of the 16 studies, 6 showed that CDK1 expression was predominantly increased in PCa samples compared with noncancer samples, while in the other 10 studies, no statistical difference in CDK1 expression was detected between PCa samples and noncancer samples (Fig. S6). The result (SMD = 0.50; 95% CI = 0.28 to 0.73; and *I*^2^ = 57.4%) indicated that CDK1 expression was significantly higher in the PCa group (1,159 samples) than in the noncancer group (491 samples) (Figure 7(a)). Due to the existing heterogeneity, a sensitivity analysis and subgroup analysis were applied to investigate the origin of the heterogeneity, but neither analysis found the origin of the heterogeneity (Figures 7(b) and 7(c)). The results of the funnel diagram showed no publication bias (Figure 7(d)). The SROC curve indicated that CDK1 had a good capability in discriminating PCa samples from noncancer samples (AUC = 0.76; sensitivity = 0.70; and specificity = 0.71) (Figure 7(e)). No publication bias was observed (Figure 7(f)). The diagnostic capacity of CDK1 in various studies is shown in Fig. S7.

#### 3.6.2. Validation of CDK1 mRNA Expression in Bone Metastatic PCa

**Figure 8:**
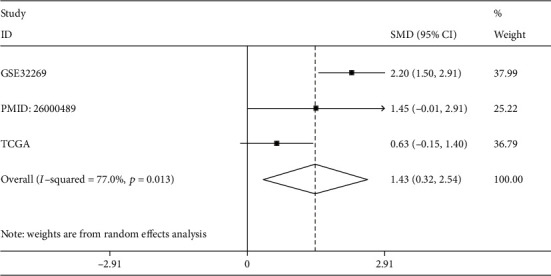
CDK1 expression level in bone metastatic PCa and nonbone metastatic PCa by calculating SMD. CDK1: cyclin-dependent kinase 1; PCa: prostate cancer; SMD: standardized mean difference; TCGA: The Cancer Genome Atlas.

According to the inclusion and exclusion criteria, a total of three studies were incorporated to verify the expression level of CDK1 in bone metastatic PCa (Table S3). All three studies showed that CDK1 was increased in bone metastatic PCa compared with nonbone metastatic PCa; however, only GSE32269 had significant statistical significance (Figs. S8A, S8C, and S8E). Next, a comprehensive evaluation of CDK1 expression was performed by calculating the SMD, which was 1.43 (95% CI = 0.32 to 2.54; *I*^2^ = 77.0%). The SMD value showed that the expression of CDK1 in bone metastatic PCa was predominantly increased compared with that in nonbone metastatic PCa (Figure 8). Three studies, including GSE32269, PMID: 26000489, and TCGA, showed that CDK1 had a good capability for discriminating between bone metastatic patients and nonbone metastatic patients, with AUC of 0.926, 0.903, and 0.785, respectively (Figs. S8B, S8D, and S8F).

#### 3.6.3. Validation of the CDK1 Protein Level in PCa

**Figure 9:**
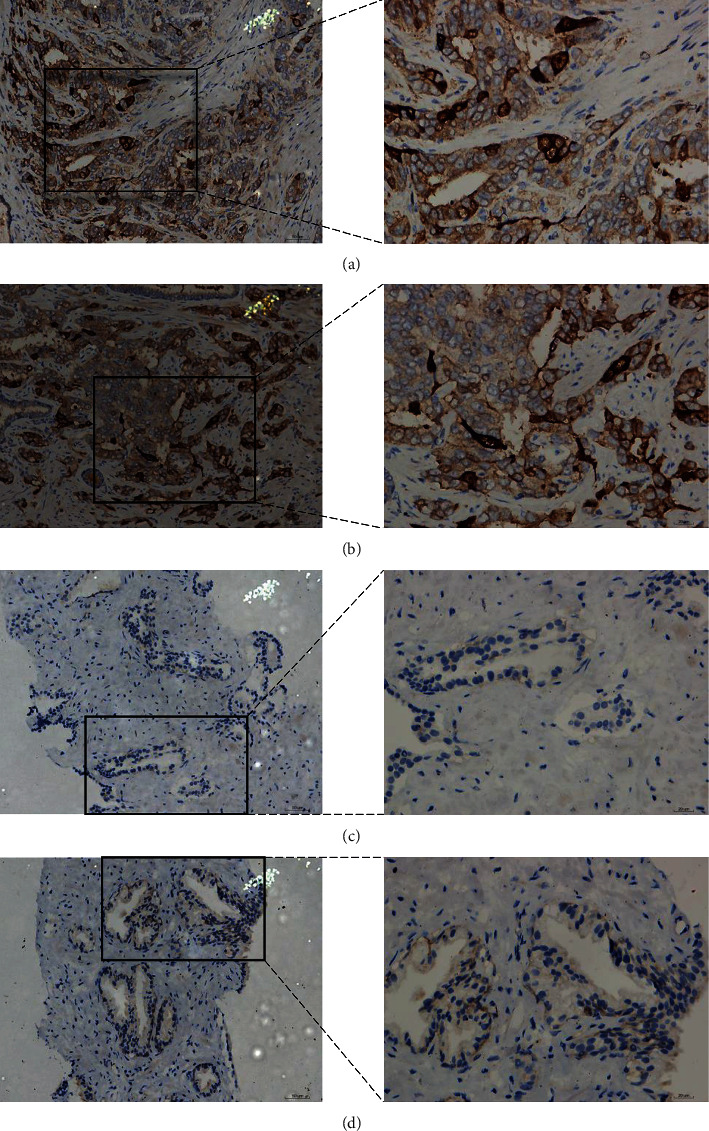
IHC staining of CDK1 protein in PCa and noncancer samples. (a, b) PCa samples stained intensely for CDK1 (magnification: ×200 lower and ×400 upper). (c, d) Noncancer samples stained at medium intensity for CDK1 (magnification: ×200 lower and ×400 upper). CDK1: cyclin-dependent kinase 1; IHC: immunohistochemistry; PCa: prostate cancer.

**Figure 10:**
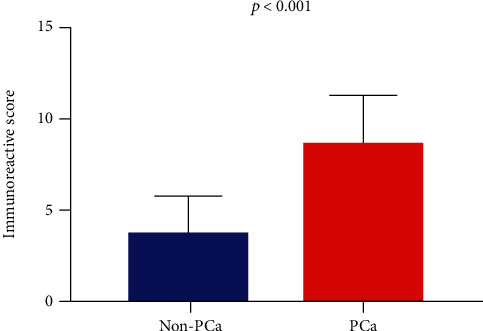
Histogram of the expression of CDK1 protein in 160 PCa samples and 61 noncancer samples by calculating the immunoreactive score. CDK1: cyclin-dependent kinase 1; PCa: prostate cancer.

The IHC results demonstrated that the expression of CDK1 in PCa samples was significantly higher than that in noncancer samples (Figures 9 and 10). In addition, we observed that upregulated CDK1 in PCa was significantly associated with pathological T, N, and M stages (Table 3). However, there was no significant correlation between CDK1 and other clinical characteristics.

### 3.7. Clinical Significance of miRNA-205 and CDK1 in PCa

**Figure 11:**
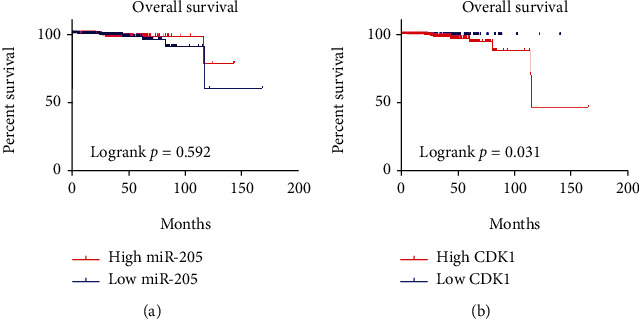
Kaplan–Meier survival curve showing the prognostic value of miRNA-205 and CDK1 in PCa using TCGA database. (a) No significant difference in OS is seen between a high and low expression of miRNA-205. (b) A high expression of CDK1 indicated a poor OS. CDK1: cyclin-dependent kinase 1; OS: overall survival; PCa: prostate cancer; TCGA: The Cancer Genome Atlas.

The association between miRNA-205 and clinical characteristics was analyzed in PCa patients using TCGA database. While miRNA-205 was significantly related to the pathological T stage and Gleason score, no statistical correlation was observed between miRNA-205 and the overall survival (OS) rate or other clinical characteristics (Table S4 and Figure 11(a)). The correlation between CDK1 and clinical characteristics in PCa patients based on TCGA database was also explored. CDK1 was significantly associated with age, pathological T and N stages, Gleason score, and recurrence (Table S5). A survival analysis showed that the PCa group with upregulated CDK1 had a shorter OS time than the PCa group with downregulated CDK1 (Figure 11(b)).

## 4. Discussion

Previous research has detected that miRNA plays a vital role in the tumorigenesis and metastasis of PCa. However, the expression and biological function of miRNA-205 in PCa remain controversial. The present study used 27 miRNA-array and miRNA-sequencing datasets containing 2,031 samples to verify the expression and diagnostic capability of miRNA-205 in PCa. In addition, the expression and distinguishing capability of miRNA-205 in bone metastatic PCa were also explored. We screened 153 miRNA-205 target genes using 3 different methods, which increased the reliability of the targets. Functional enrichment analysis was applied to clarify the biological mechanism of miRNA-205 in PCa. CDK1 had the highest connectivity in the PPI network analysis and was screened as one of the hub genes. In PCa, the mRNA expression level of CDK1 was verified using 1,650 samples from 16 datasets. Meanwhile, IHC was used to verify the CDK1 protein expression level. The clinicopathological significance of miRNA-205 and CDK1 in PCa were also explored. This study is the first extensive analysis of the significance of miRNA-205 and CDK1 in PCa.

Our study found that PCa samples had a lower expression of miRNA-205 compared with noncancer samples, which was inconsistent with the results of studies by Osipov et al. and Stephan et al. Osipov et al. tested the miRNA-205 expression in 48 PCa patient blood samples and 47 healthy volunteer blood samples and found that miRNA-205 expression was significantly higher in PCa patients than in controls. The discrepancies between their results and those of the present study may be due to small sample sizes (*n* < 50) and the different detection methods. In the study by Stephan et al., urine sediment from 38 PCa patients and 38 noncancer patients was collected to assess the expression of miRNA-205 in PCa, and no difference in miRNA-205 expression was detected between the two groups. Stephan et al. explained that the possible reason for this was that they only measured the miRNA-205 in urine sediment and excluded that from exosomes. The AUC value of the SROC curve of the miRNA-205 was 0.92, which indicated that miRNA-205 had an excellent capability in differentiating between PCa samples and noncancer samples. In addition, miRNA-205 was significantly associated with the pathological T stage and Gleason score. The above results showed that miRNA-205 might be a tumor suppressor gene involved in PCa.

Currently, only one study has reported the expression of miRNA-205 in bone metastatic PCa. Guo et al. tested 34 bone metastatic PCa samples and 38 nonbone metastatic PCa samples, and they found that the expression of miRNA-205 in bone metastatic PCa samples was lower compared with that in nonbone metastatic PCa samples [29], consistent with our result. In addition, miRNA-205 had a good capability in distinguishing between bone metastatic PCa samples and nonbone metastatic PCa. However, large sample cohorts are needed to further verify our results.

At present, the mechanism of PCa tumorigenesis and bone metastasis is not clear. GO and KEGG analyses were performed to elucidate the role of miRNA-205 targets in PCa. Some important GO and KEGG entries have been reported that play important roles in PCa. For example, the expression of FZD8 was found to be significantly higher in bone metastatic PCa samples and promoted the occurrence of bone metastatic PCa samples by activating the Wnt signaling pathway [30]. Both miRNA-182 and miRNA-200 negatively regulated the expression of GNA13, thereby reducing the invasion and migration capability of PCa [31]. TMSG1 regulated ATPase activity and inhibited the invasion and metastasis of PCa cells [32]. Long noncoding RNA LINP1 promoted the progression of PCa by regulating the p53 signaling pathway [33]. Therefore, it is meaningful to further study the role of miRNA-205 targets in PCa.

Previous studies have reported that miRNA-205 may affect the progression and metastasis of PCa by targeting HMGB3, c-SRC, and CENPF [21–23]. However, no other targets of miRNA-205 have been reported. In this study, CDK1 was selected as the hub gene and the most likely to be targeted by miRNA-205. CDK1 is a member of the serine/threonine protein kinase family, which is necessary for G1/S and G2/M phase transitions in the eukaryotic cell cycle [34]. CDK1 plays an important role in a variety of malignancies, including PCa. In human melanoma, the interaction between CDK1 and SOX2 could promote tumorigenesis [35]. CDK1 was significantly increased in ovarian cancer, and knocking down CDK1 reduced the growth of ovarian cancer cells [36]. The upregulation of CDK1 was associated with poor survival in patients with renal clear cell carcinoma [37]. MicroRNA-1271 was increased in endometrial cancer and reduced proliferation by inhibiting CDK1 expression [38]. In PCa, CDK1 phosphorylated the androgen receptor (AR) and promoted PCa cell proliferation, while CDK1 inhibitors reduced AR phosphorylation and protein expression in PCa cells [39, 40]. Decreased levels of CDK1 reduced tumor growth activity, and increased levels of CDK1 promoted PCa cells to develop in the direction of G2/M, which may be the cause of everolimus resistance [41]. In Sarwar et al.'s research, CDK1 complexes with AR-V7 and PIP5K1*α* to accelerate PCa growth and metastasis [42]. CDK1 was overexpressed in metastatic castration-resistant PCa versus hormone-sensitive PCa and could be used as a prognostic indicator of PCa [43].

In our study, the mRNA level and protein expression level of CDK1 were significantly higher in PCa samples compared with noncancer samples, according to the SMD and IHC. The high expression of CDK1 can distinguish PCa samples from noncancer samples with 70% sensitivity and 71% specificity. CDK1 expression was further increased in bone metastatic PCa samples compared with nonbone metastatic PCa samples, and it had a good capability in distinguishing between the two groups. The results of our study showed CDK1 was significantly associated with pathological T and N stages, recurrence, and overall survival. The above results indicate that CDK1 plays a vital role in PCa occurrence and bone metastasis and might be a therapeutic target for PCa.

Although we obtained valuable findings, this study does have some limitations. First, there was significant heterogeneity, which would reduce the reliability of our results. Even though we tried to solve this problem, the results of subgroup analysis and sensitivity analysis failed to identify the source of heterogeneity. The following factors may be sources of heterogeneity. (1) Datasets were collected from 10 different countries, including the USA, Turkey, Spain, Mexico, Italy, Iran, Germany, Finland, China, and Brazil. (2) The control group had different sources, including normal prostate tissues, paracancerous tissues, benign prostatic hyperplasia tissues, healthy body fluids, and normal cell lines. (3) There were different platforms in GEO microarrays, variant experimental methods, discrepant research designs, and different histological types of PCa. (4) The sample sizes ranged from 8 to 550. Therefore, subgroup analysis based on large-scale clinical trials is needed to find the source of heterogeneity. Second, we have determined the expression and clinical significance of miRNA-205 and CDK1 in PCa; however, the biological functions of miRNA-205 and CDK1 in PCa and the interaction between miRNA-205 and CDK1 need further to be verified through both *in vitro* and *in vivo* experiments.

## 5. Conclusions

Overall, the present study demonstrated that miRNA-205 is downregulated in both PCa and bone metastatic PCa. In addition, miRNA-205 may be involved in the carcinogenesis and bone metastasis of PCa by negatively regulating CDK1 expression.

## Figures and Tables

**Table 1:** The miRNA-205 expression in PCa samples and noncancer samples based on miRNA-array and miRNA-sequencing data.

Study	Country	Year	Sample type	PCa			Non-PCa		
				*N*	*M*	SD	*N*	*M*	SD
GSE14857	Germany	2009	Tissue	12	7.931	3.160	12	12.062	0.522
GSE18671	Germany	2011	Tissue	14	6.968	0.447	6	9.256	0.691
GSE21036	USA	2010	Tissue	114	9.665	3.657	28	12.598	0.658
GSE23022	Germany	2010	Tissue	20	3.229	1.441	20	4.161	1.290
GSE34932	China	2012	Tissue	8	3.096	1.970	8	9.750	0.635
GSE36802	USA	2013	Tissue	21	9.265	3.004	21	12.858	0.424
GSE54516	Germany	2014	Tissue	51	18.171	2.421	48	15.635	0.571
GSE60117	Italy	2014	Tissue	56	9.805	1.628	21	10.746	0.425
GSE64318	USA	2014	Tissue	27	9.229	0.767	27	8.789	2.592
GSE76260	Italy	2015	Tissue	32	14.291	0.268	32	14.490	0.182
GSE2564	USA	2012	Tissue	6	11.218	1.529	8	12.186	0.070
GSE8126	USA	2012	Tissue	60	12.176	1.413	16	11.380	1.519
GSE26367	USA	2012	Tissue	173	13.140	1.540	11	13.978	1.996
GSE46738	Brazil	2013	Tissue	53	10.298	1.147	4	10.084	0.620
GSE89193	USA	2016	Tissue	49	2.793	2.087	49	4.676	1.873
GSE48430	USA	2014	Tissue	10	29.457	6.453	10	25.511	3.213
E-MTAB-408	Finland	2012	Tissue	42	9.229	3.030	12	6.782	3.191
TCGA	NA	NA	Tissue	498	8.719	2.211	52	9.888	1.674
Nassim Ghorbanmehr	Iran	2019	Body fluid	17	6.299	2.214	28	8.427	2.552
GSE49298	Turkey	2013	Body fluid	4	4.906	1.248	4	5.208	0.657
GSE39314	Mexico	2012	Body fluid	9	5.115	3.901	8	6.606	2.326
GSE45604	Spain	2014	Body fluid	50	19.920	3.388	10	22.848	3.259
GSE61741	Germany	2014	Body fluid	65	5.583	1.902	35	4.887	2.209
GSE31568	Germany	2011	Body fluid	23	5.765	0.658	70	5.109	1.765
GSE97901	USA	2017	Body fluid	32	1.661	0.518	13	1.945	0.753
GSE16512	USA	2009	Body fluid	6	7.411	1.428	14	6.893	0.882
GSE17317	Germany	2009	Cell line	9	10.494	1.470	3	12.680	0.126

*M*: mean; *N*: number; PCa: prostate cancer; SD: standard deviation; TCGA: The Cancer Genome Atlas.

**Table 2:** CDK1 expression in PCa samples and noncancer samples based on mRNA-array and mRNA-sequencing data.

Study	Country	Year	Sample type	PCa			Non-PCa		
				*N*	*M*	SD	*N*	*M*	SD
GSE6919	USA	2007	Tissue	90	4.854	0.889	81	4.096	0.845
GSE104749	China	2017	Tissue	4	8.876	0.846	4	5.389	0.539
GSE73397	China	2015	Tissue	3	9.600	0.086	3	9.393	0.097
GSE69223	Germany	2015	Tissue	15	0.141	0.994	15	−0.250	0.761
GSE94767	UK	2017	Tissue	185	4.376	0.697	33	3.937	0.366
GSE60329	Italy	2014	Tissue	108	−0.063	1.089	28	−0.046	0.571
GSE72220	USA	2015	Tissue	57	−0.101	0.090	90	−0.127	0.106
GSE46602	Denmark	2013	Tissue	34	2.767	0.418	14	2.534	0.247
GSE38043	USA	2012	Body fluid	3	6.000	0.124	3	6.655	0.754
GSE32982	Finland	2011	Tissue	6	5.582	0.447	3	5.473	0.119
GSE35988	USA	2012	Tissue	76	1.829	1.676	12	0.008	0.584
GSE28204	China	2011	Tissue	4	8.031	6.381	4	6.381	0.762
GSE32448	USA	2011	Tissue	40	5.861	0.756	40	5.740	0.811
GSE26910	Italy	2011	Tissue	6	3.354	0.323	6	3.279	0.181
GSE12378	UK	2008	Tissue	36	4.128	0.494	3	3.973	0.427
TCGA+GTEx	NA	NA	Tissue	492	1.461	0.754	152	1.048	0.570

CDK1: cyclin-dependent kinase 1; GTEx: The Genotype-Tissue Expression; *M*: mean; *N*: number; PCa: prostate cancer; SD: standard deviation; TCGA: The Cancer Genome Atlas.

**Table 3:** Association between CDK1 expression and clinicopathological parameters in PCa samples based on IHC.

Clinicopathological parameters	*N*	CDK1 expression		*t* test	
		*M*	SD	*t* value	*p* value
Group					
Noncancer	61	3.689	1.937	-15.895	≤0.001
Cancer	160	8.811	2.595		
Age (years)					
<60	16	9.867	2.560	1.664	0.098
≥60	144	8.703	5.583		
Pathological T stage					
T1+T2	146	8.545	2.519	-6.346	≤0.001
T3+T4	14	11.571	1.604		
N stage					
N0	155	8.708	2.572	-15.887	≤0.001
N1	5	12.000	0.000		
M stage					
M0	158	8.791	2.591	-15.566	≤0.001
M1	2	12.000	0.000		
Gleason score					
≤7	90	8.921	2.647	0.602	0.548
≥8	70	8.671	2.541		

Note: IHC: immunohistochemistry; CDK1: cyclin-dependent kinase 1; *M*: mean; *N*: number; PCa: prostate cancer.

## Data Availability

The raw data is available at the GEO, ArrayExpress, Oncomine, SRA, and TCGA databases.

## Supplementary Materials

Supplementary 1 Supplemental Figure S1: Expression expression level of miRNA-205 in PCa tissues. PCa: prostate cancer; TCGA: The Cancer Genome Atlas.

Supplementary 2 Supplemental Figure S2: Expression expression level of miRNA-205 in PCa body fluids and cell lines. PCa: prostate cancer.

Supplementary 3 Supplemental Figure S3: Diagnostic diagnostic capability of miRNA-205 in PCa tissues. PCa: prostate cancer.

Supplementary 4 Supplemental Figure S4: Diagnostic diagnostic capability of miRNA-205 in PCa body fluids and cell lines. PCa: prostate cancer.

Supplementary 5 Supplemental Figure S5: Expression expression levels and diagnostic capability of miRNA-205 in bone metastatic PCa and non–bone metastatic PCa. (A, B) GSE21036. (C, D) GSE26964. (E, F) TCGA. AUC: area under the curve; BM: bone metastatic; nBM: non–bone metastatic; PCa: prostate cancer; TCGA: The Cancer Genome Atlas.

Supplementary 6 Supplemental Figure S6: Expression expression level of CDK1 in PCa. CDK1: cyclin-dependent kinase 1; PCa: prostate cancer.

Supplementary 7 Supplemental Figure S7: Diagnostic diagnostic capability of CDK1 in PCa. AUC: area under the curve; CDK1: cyclin-dependent kinase 1; PCa: prostate cancer.

Supplementary 8 Supplemental Figure S8: Expression expression levels and diagnostic capability of CDK1 in bone metastatic PCa and non–bone metastatic PCa. (A, B) GSE32269. (C, D) PMID: 26000489. (E, F) TCGA. AUC: area under the curve; BM: bone metastatic; CDK1: cyclin-dependent kinase 1; nBM: non–bone metastatic; PCa: prostate cancer; TCGA: The Cancer Genome Atlas.

Supplementary 9 Supplemental Table S1: The the miRNA-205 expression in bone metastatic PCa samples and non–bone metastatic PCa samples.

Supplementary 10 Supplemental Table S2: Significant significant terms of Gene Ontology (GO) and Kyoto Encyclopedia of Genes and Genomes (KEGG).

Supplementary 11 Supplemental Table S3: CDK1 expression in bone metastatic PCa samples and non–bone metastatic PCa samples.

Supplementary 12 Supplemental Table S4: Association association between miRNA-205 expression and clinicopathological parameters in PCa samples based on TCGA database.

Supplementary 13 Supplemental Table S5: Association association between CDK1 expression and clinicopathological parameters in PCa samples based on TCGA database.
