# Supplementary figures and images for: Downregulation of miRNA-205 Expression and Biological Mechanism in Prostate Cancer Tumorigenesis and Bone Metastasis
Source: Biomed Res Int. 2020 Oct 29;2020:6037434. doi: 10.1155/2020/6037434 (PMC7646560; doi:10.1155/2020/6037434)

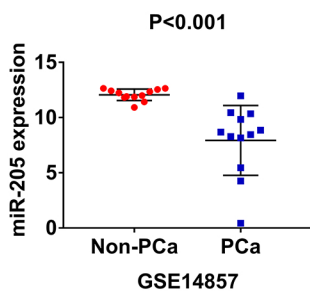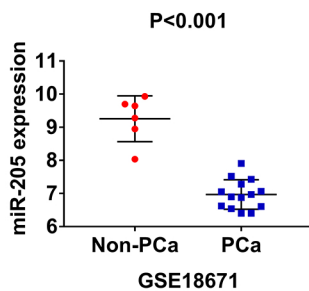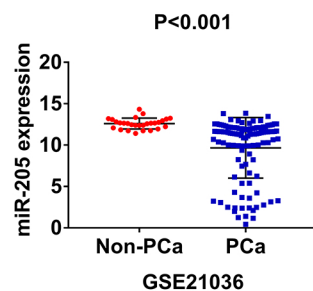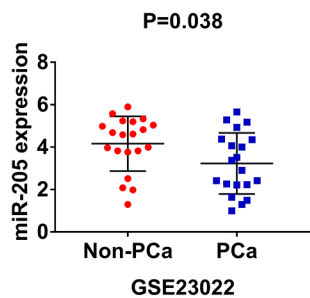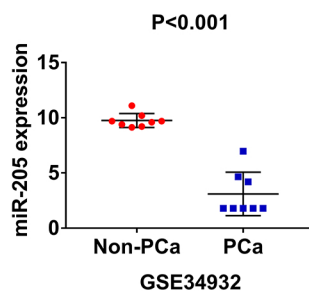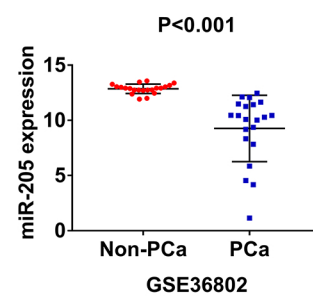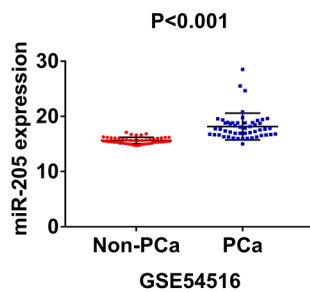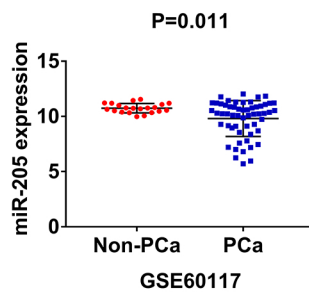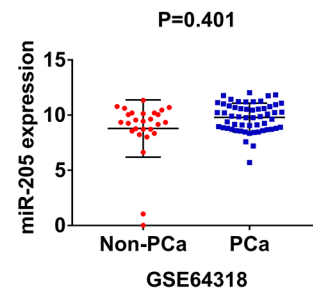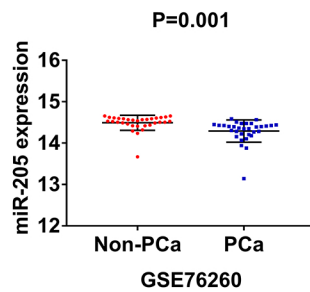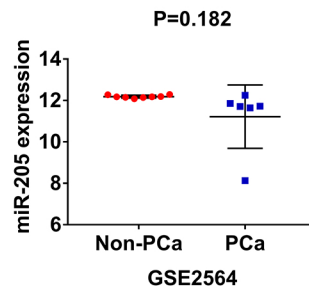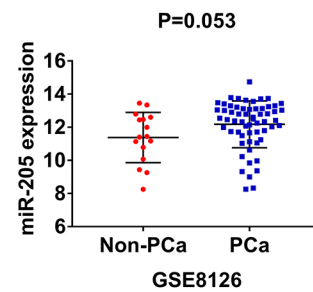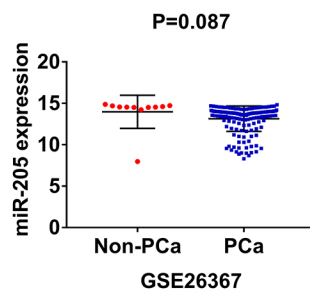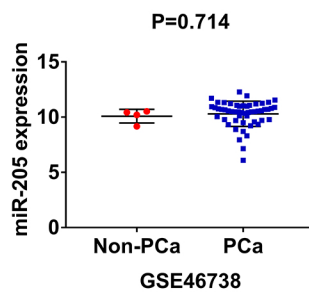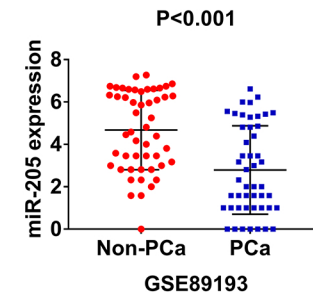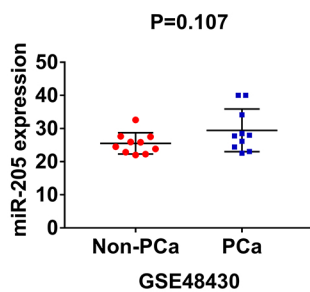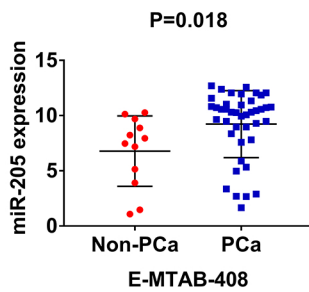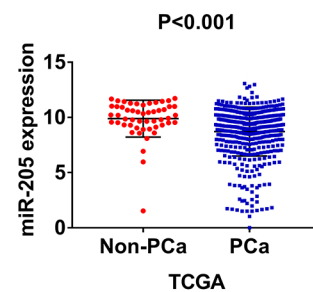

Supplement: Supplementary 1 — Supplemental Figure S1: Expression expression level of miRNA-205 in PCa tissues. PCa: prostate cancer; TCGA: The Cancer Genome Atlas. [file 6037434.f1.pdf]

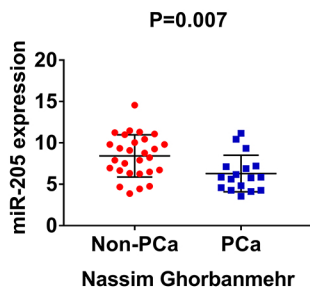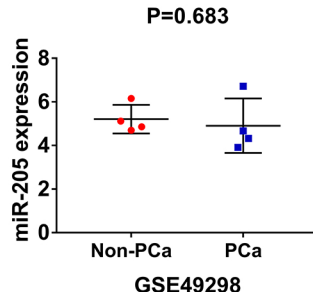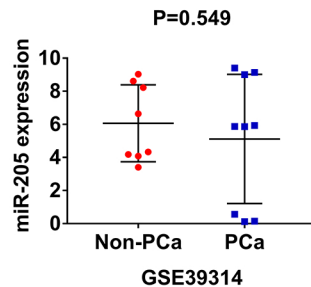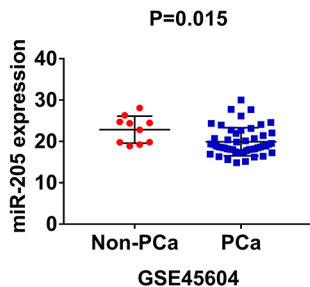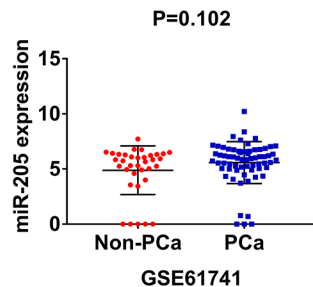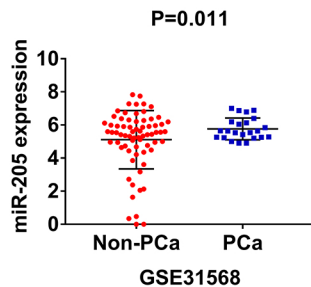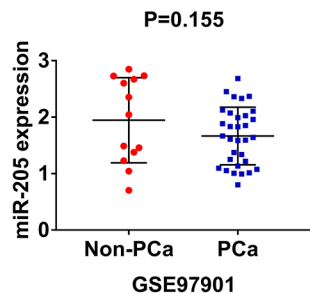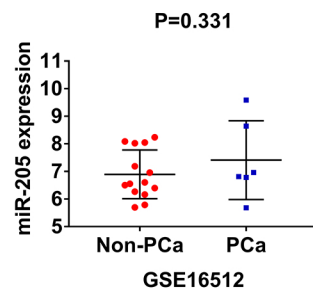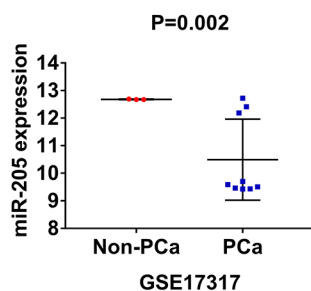

Supplement: Supplementary 2 — Supplemental Figure S2: Expression expression level of miRNA-205 in PCa body fluids and cell lines. PCa: prostate cancer. [file 6037434.f2.pdf]

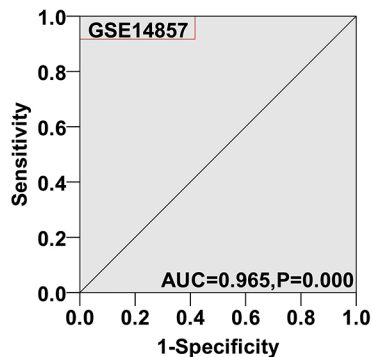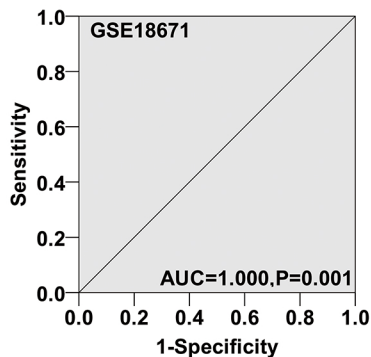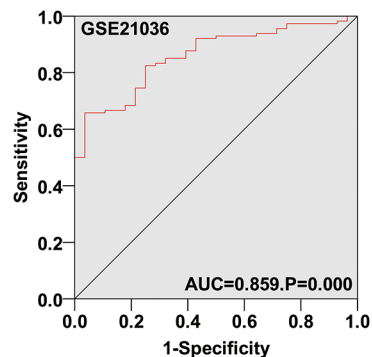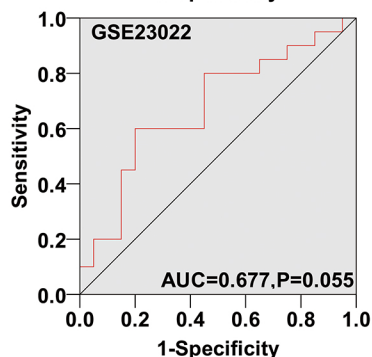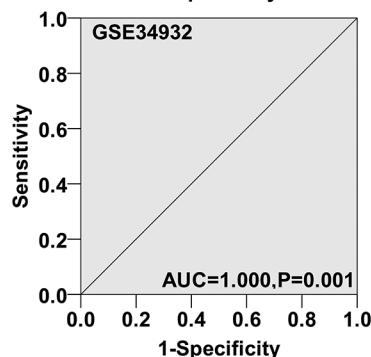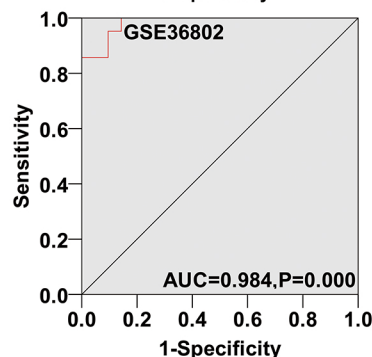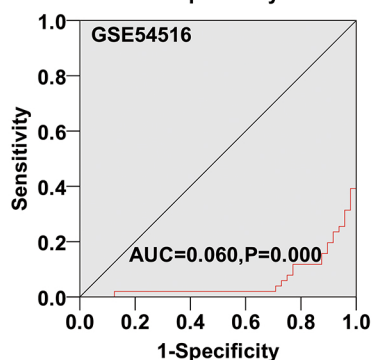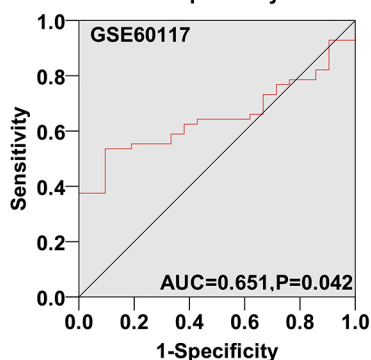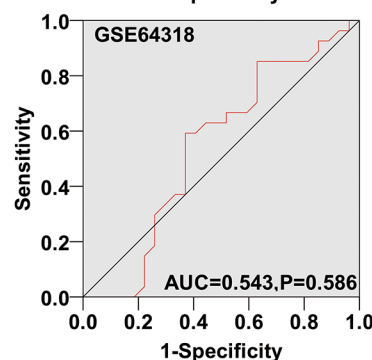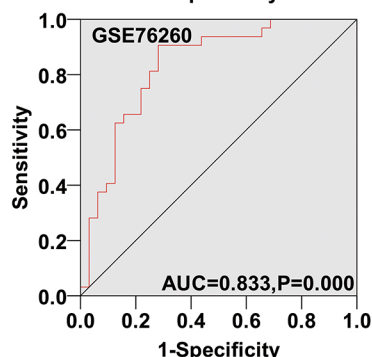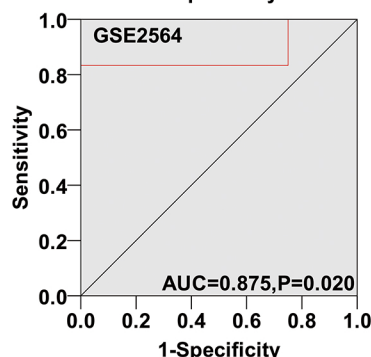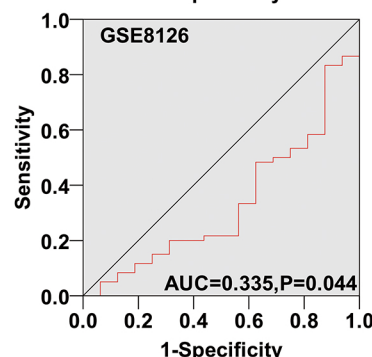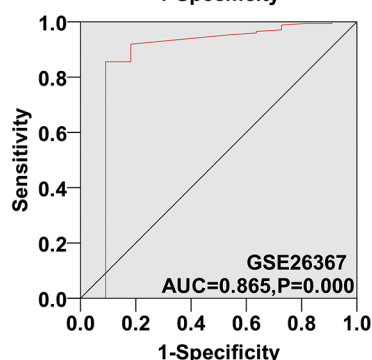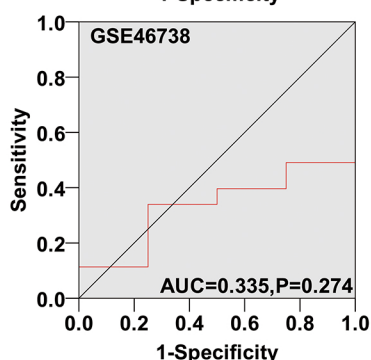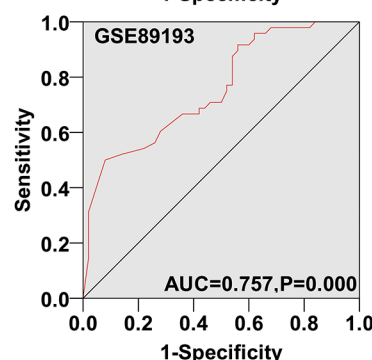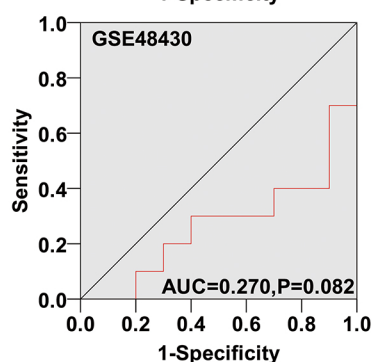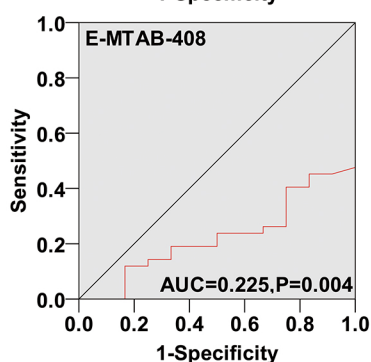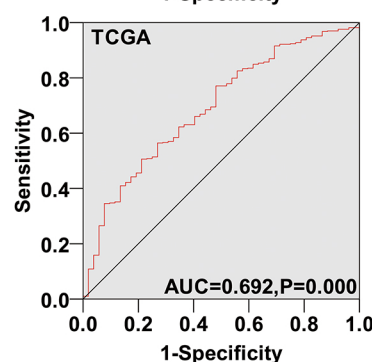

Supplement: Supplementary 3 — Supplemental Figure S3: Diagnostic diagnostic capability of miRNA-205 in PCa tissues. PCa: prostate cancer. [file 6037434.f3.pdf]

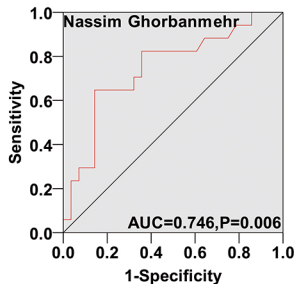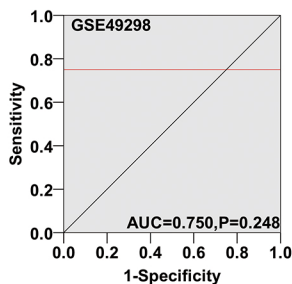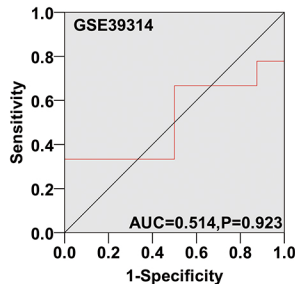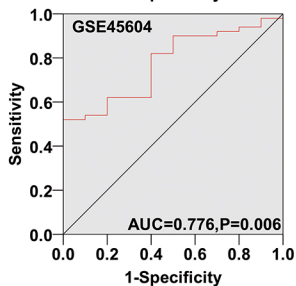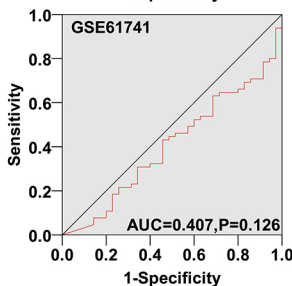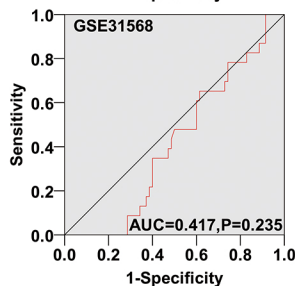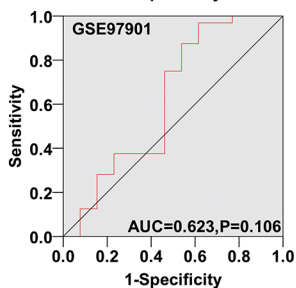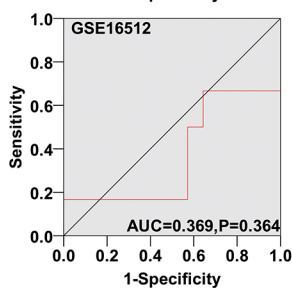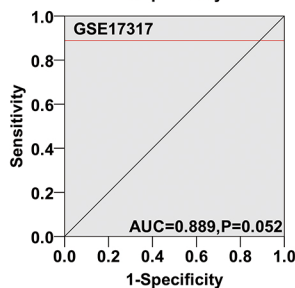

Supplement: Supplementary 4 — Supplemental Figure S4: Diagnostic diagnostic capability of miRNA-205 in PCa body fluids and cell lines. PCa: prostate cancer. [file 6037434.f4.pdf]

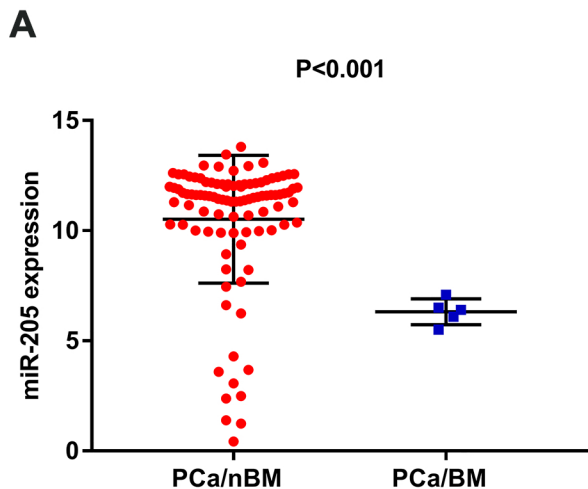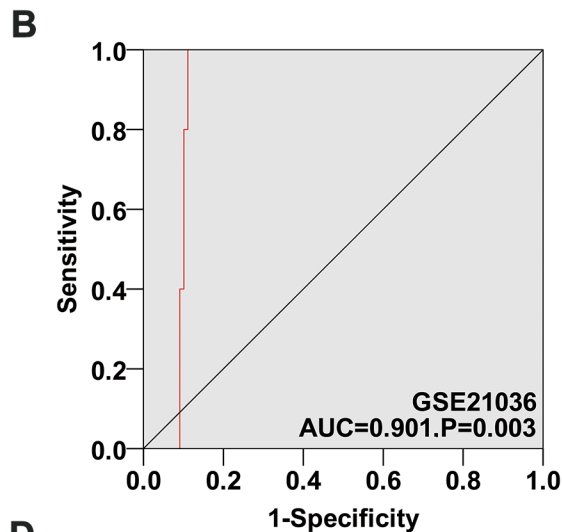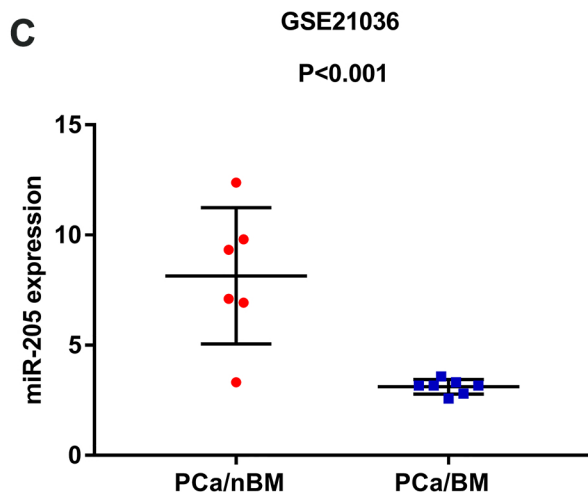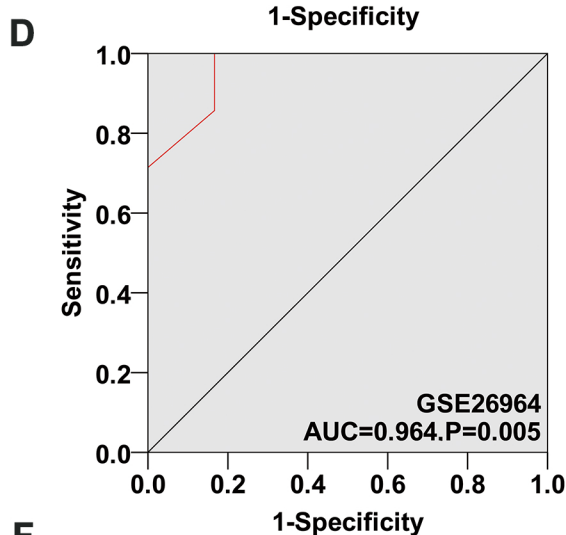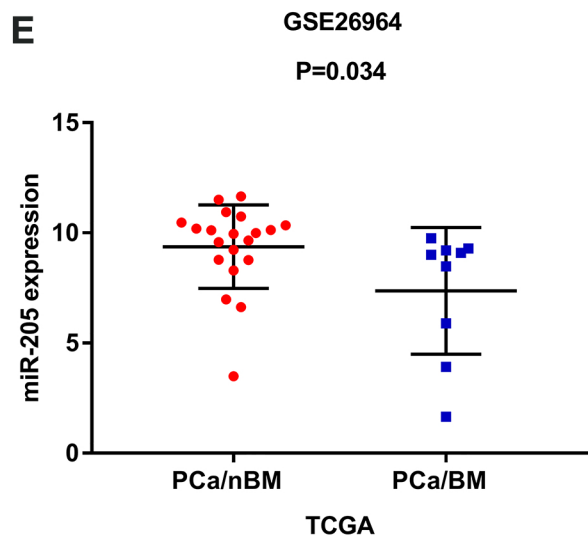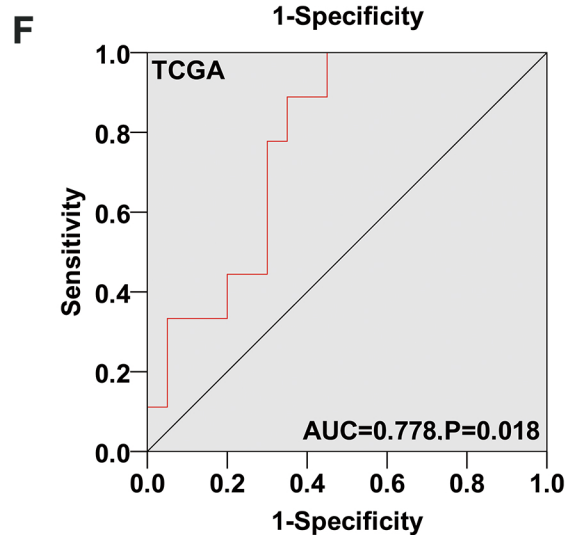

Supplement: Supplementary 5 — Supplemental Figure S5: Expression expression levels and diagnostic capability of miRNA-205 in bone metastatic PCa and non–bone metastatic PCa. (A, B) GSE21036. (C, D) GSE26964. (E, F) TCGA. AUC: area under the curve; BM: bone metastatic; nBM: non–bone metastatic; PCa: prostate cancer; TCGA: The Cancer Genome Atlas. [file 6037434.f5.pdf]

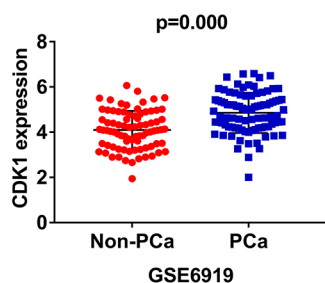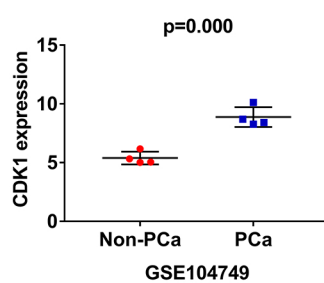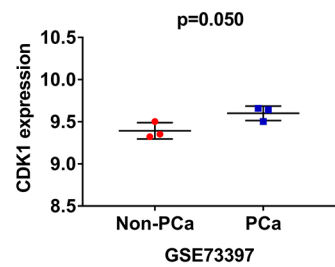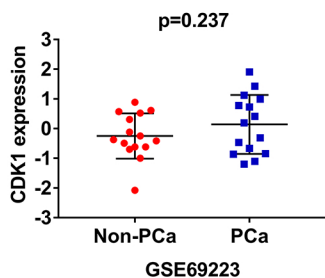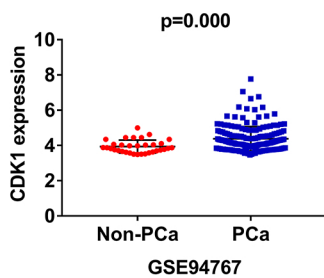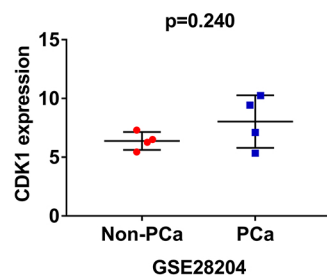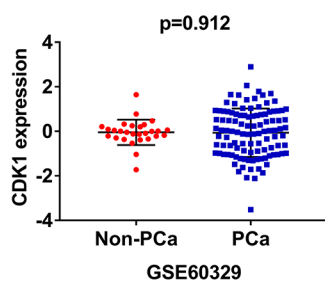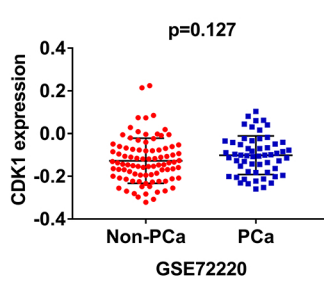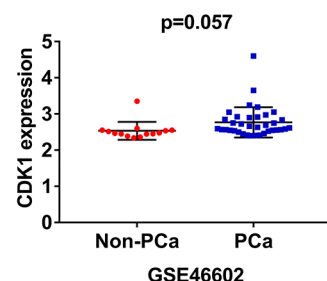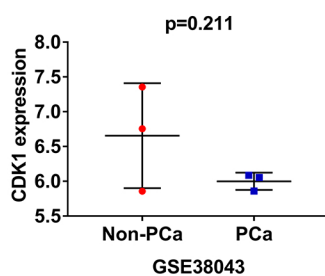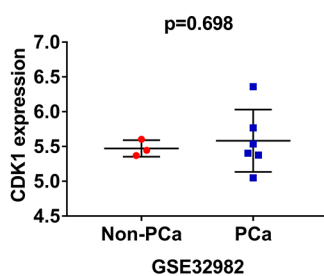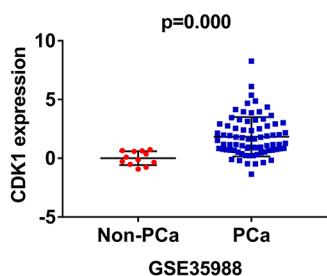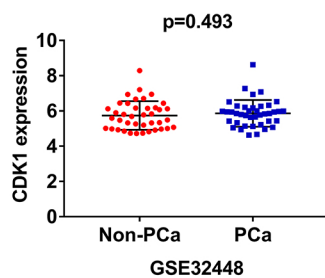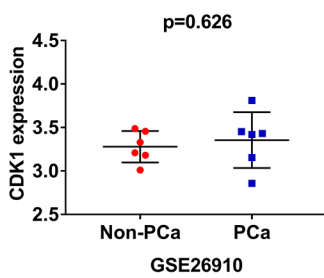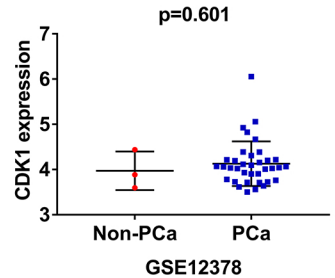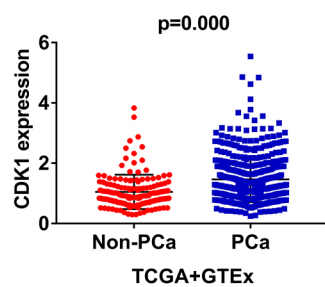

Supplement: Supplementary 6 — Supplemental Figure S6: Expression expression level of CDK1 in PCa. CDK1: cyclin-dependent kinase 1; PCa: prostate cancer. [file 6037434.f6.pdf]

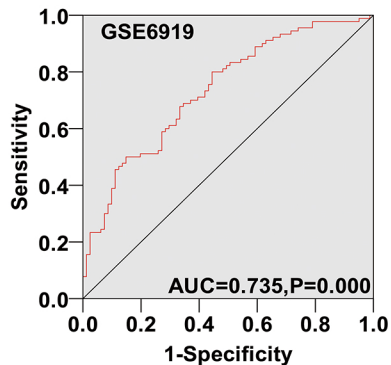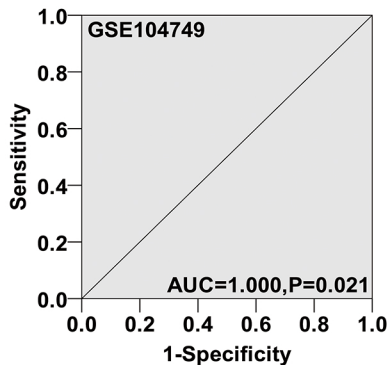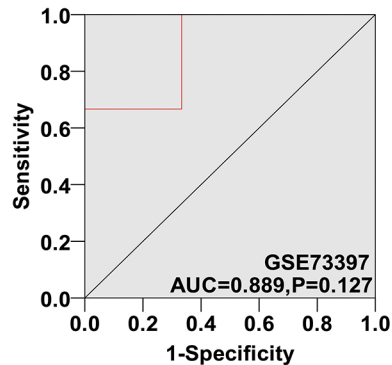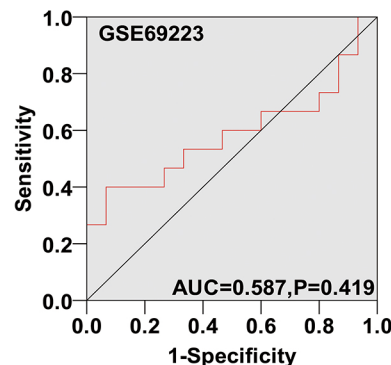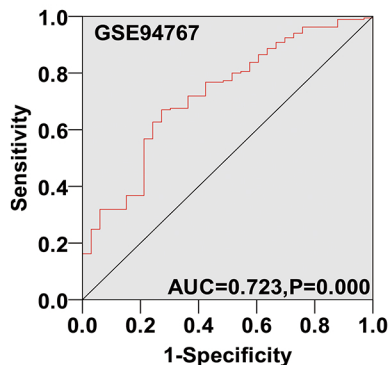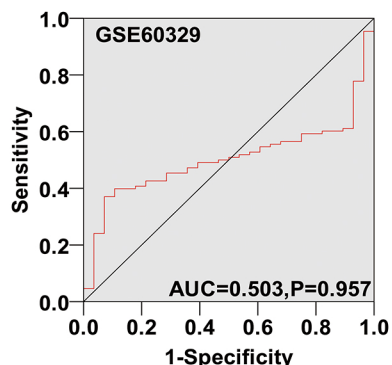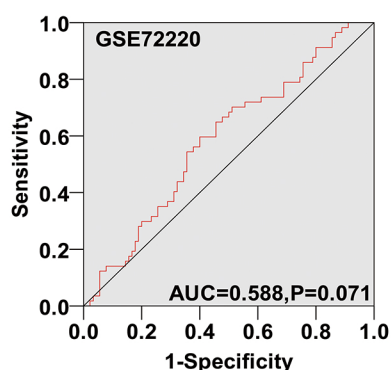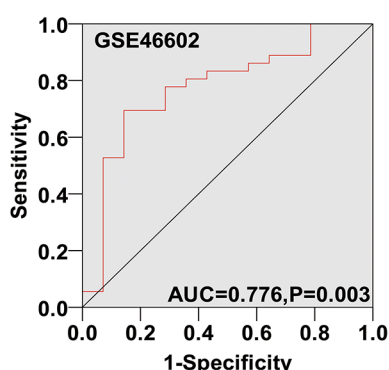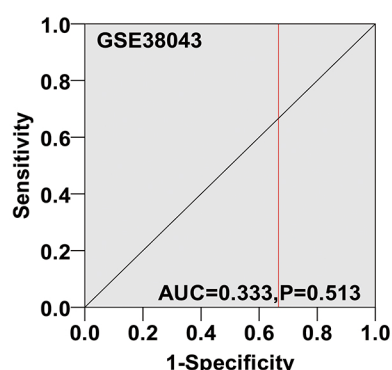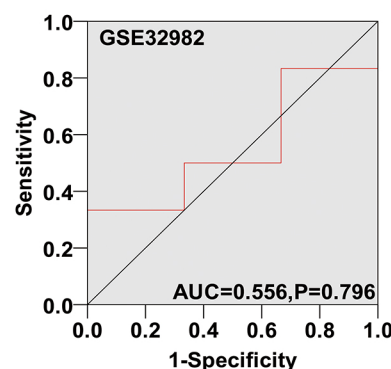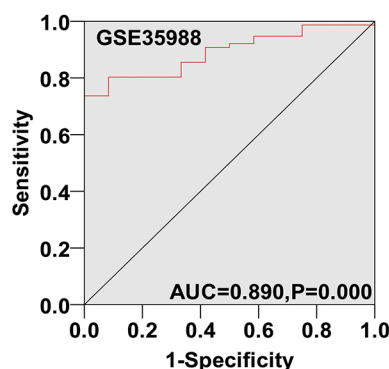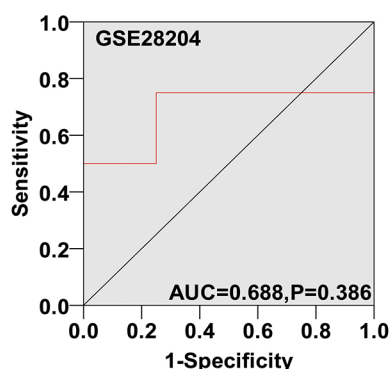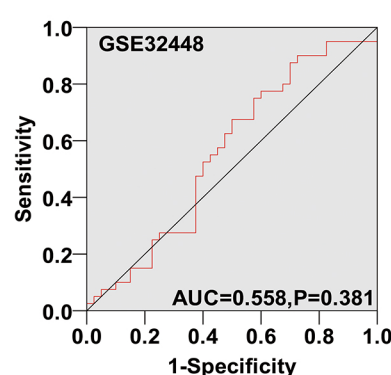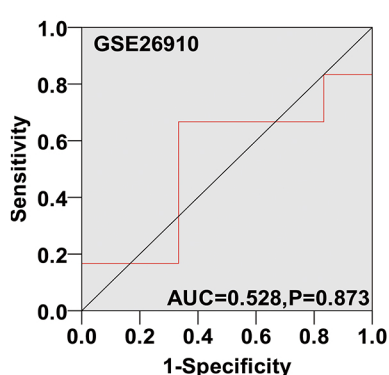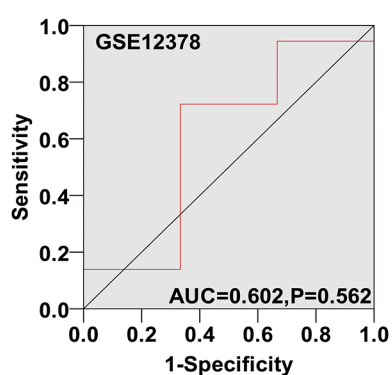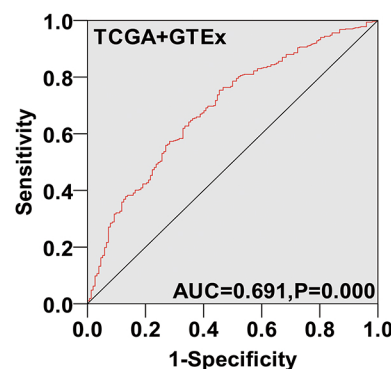

Supplement: Supplementary 7 — Supplemental Figure S7: Diagnostic diagnostic capability of CDK1 in PCa. AUC: area under the curve; CDK1: cyclin-dependent kinase 1; PCa: prostate cancer. [file 6037434.f7.pdf]

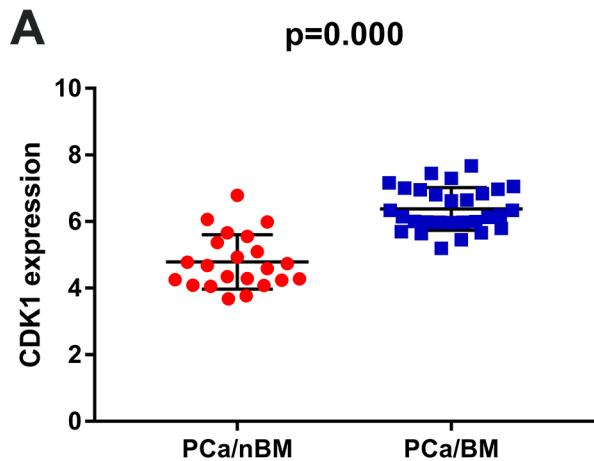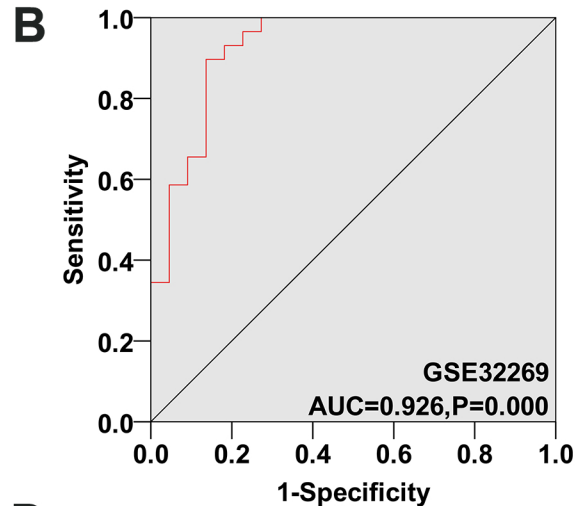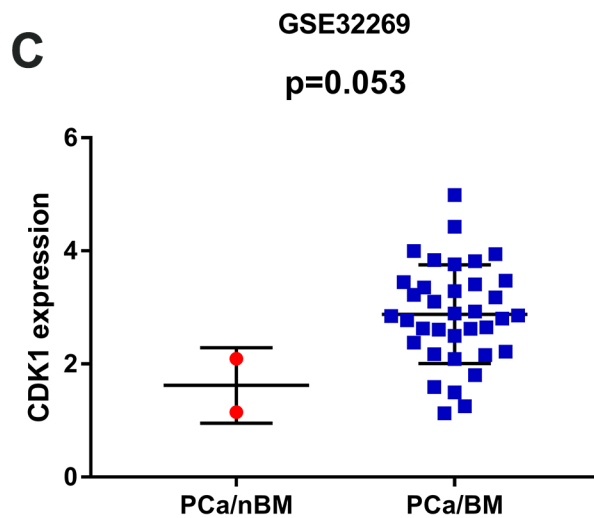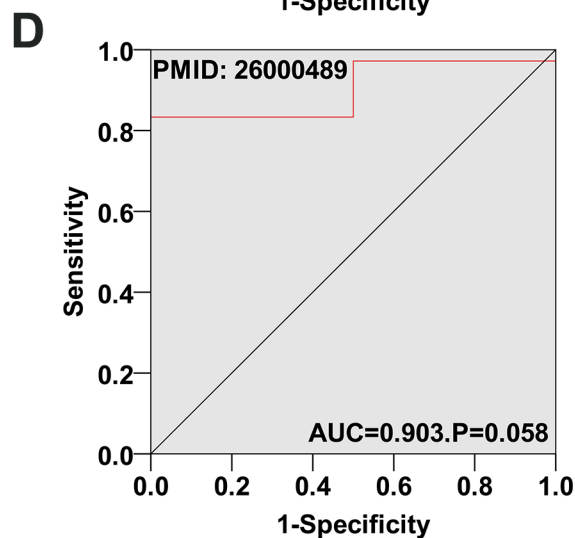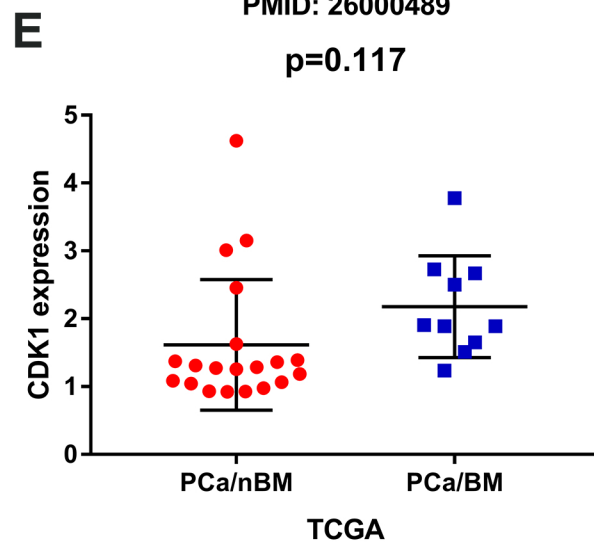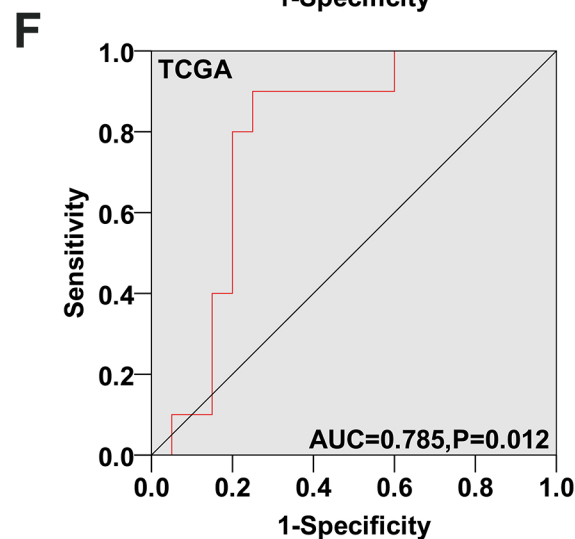

Supplement: Supplementary 8 — Supplemental Figure S8: Expression expression levels and diagnostic capability of CDK1 in bone metastatic PCa and non–bone metastatic PCa. (A, B) GSE32269. (C, D) PMID: 26000489. (E, F) TCGA. AUC: area under the curve; BM: bone metastatic; CDK1: cyclin-dependent kinase 1; nBM: non–bone metastatic; PCa: prostate cancer; TCGA: The Cancer Genome Atlas. [file 6037434.f8.pdf]
