# Supplementary material for: Downregulation of miRNA-205 Expression and Biological Mechanism in Prostate Cancer Tumorigenesis and Bone Metastasis
Source: Biomed Res Int. 2020 Oct 29;2020:6037434. doi: 10.1155/2020/6037434 (PMC7646560; doi:10.1155/2020/6037434)
Supplement: Supplementary 9 — Supplemental Table S1: The the miRNA-205 expression in bone metastatic PCa samples and non–bone metastatic PCa samples. [file 6037434.f9.docx]

Supplemental Table S1. The miRNA-205 expression in bone metastatic PCa samples and non–bone metastatic PCa samples.

| Study | Country | Year | Sample | PCa/BM | | |  | PCa/nBM | | |
| --- | --- | --- | --- | --- | --- | --- | --- | --- | --- | --- |
|  |  |  | type | N | M | SD |  | N | M | SD |
| GSE21036 | USA | 2010 | Tissue | 5 | 6.361 | 4.385 |  | 99 | 10.524 | 2.900 |
| GSE26964 | China | 2011 | Tissue | 7 | 3.116 | 0.329 |  | 6 | 8.147 | 3.097 |
| TCGA | NA | NA | Tissue | 9 | 7.369 | 2.881 |  | 20 | 9.375 | 2.881 |

BM: bone metastatic; M: mean; N: number; nBM: non–bone metastatic; PCa: prostate cancer; SD: standard deviation; TCGA: The Cancer Genome Atlas.
