# Supplementary material for: Downregulation of miRNA-205 Expression and Biological Mechanism in Prostate Cancer Tumorigenesis and Bone Metastasis
Source: Biomed Res Int. 2020 Oct 29;2020:6037434. doi: 10.1155/2020/6037434 (PMC7646560; doi:10.1155/2020/6037434)
Supplement: Supplementary 10 — Supplemental Table S2: Significant significant terms of Gene Ontology (GO) and Kyoto Encyclopedia of Genes and Genomes (KEGG). [file 6037434.f10.docx]

Supplemental Table S2. Significant terms of Gene Ontology (GO) and Kyoto Encyclopedia of Genes and Genomes (KEGG).

| Category | Term | Count | Ratio | p value |
| --- | --- | --- | --- | --- |
| GOTERM_BP_DIRECT | GO:0007059~chromosome segregation | 5 | 0.020277 | 0.000807 |
| GOTERM_BP_DIRECT | GO:0006351~transcription, DNA-templated | 10 | 0.040555 | 0.001778 |
| GOTERM_BP_DIRECT | GO:0007080~mitotic metaphase plate congression | 4 | 0.016222 | 0.001896 |
| GOTERM_BP_DIRECT | GO:0007067~mitotic nuclear division | 4 | 0.016222 | 0.006089 |
| GOTERM_BP_DIRECT | GO:0032467~positive regulation of cytokinesis | 3 | 0.012166 | 0.01854 |
| GOTERM_BP_DIRECT | GO:0032877~positive regulation of DNA endoreduplication | 2 | 0.008111 | 0.019063 |
| GOTERM_BP_DIRECT | GO:0007018~microtubule-based movement | 4 | 0.016222 | 0.021507 |
| GOTERM_BP_DIRECT | GO:0060548~negative regulation of cell death | 3 | 0.012166 | 0.027312 |
| GOTERM_BP_DIRECT | GO:0060070~canonical Wnt signaling pathway | 4 | 0.016222 | 0.028402 |
| GOTERM_CC_DIRECT | GO:0005876~spindle microtubule | 5 | 0.020277 | 0.000183 |
| GOTERM_CC_DIRECT | GO:0051233~spindle midzone | 4 | 0.016222 | 0.000364 |
| GOTERM_CC_DIRECT | GO:0000775~chromosome, centromeric region | 4 | 0.016222 | 0.000523 |
| GOTERM_CC_DIRECT | GO:0030496~midbody | 5 | 0.020277 | 0.005796 |
| GOTERM_CC_DIRECT | GO:0015629~actin cytoskeleton | 5 | 0.020277 | 0.024831 |
| GOTERM_CC_DIRECT | GO:0097149~centralspindlin complex | 2 | 0.008111 | 0.026702 |
| GOTERM_CC_DIRECT | GO:0032133~chromosome passenger complex | 2 | 0.008111 | 0.04411 |
| GOTERM_CC_DIRECT | GO:0034362~low-density lipoprotein particle | 2 | 0.008111 | 0.04411 |
| GOTERM_CC_DIRECT | GO:0000940~condensed chromosome outer kinetochore | 2 | 0.008111 | 0.048697 |
| GOTERM_MF_DIRECT | GO:0004012~phospholipid-translocating ATPase activity | 3 | 0.012166 | 0.008294 |
| GOTERM_MF_DIRECT | GO:0004487~methylenetetrahydrofolate dehydrogenase activity | 2 | 0.008111 | 0.025856 |
| GOTERM_MF_DIRECT | GO:0004477~methenyltetrahydrofolate cyclohydrolase activity | 2 | 0.008111 | 0.025856 |
| GOTERM_MF_DIRECT | GO:0001965~G-protein alpha-subunit binding | 2 | 0.008111 | 0.034327 |
| GOTERM_MF_DIRECT | GO:0003682~chromatin binding | 7 | 0.028388 | 0.041746 |
| GOTERM_MF_DIRECT | GO:0016887~ATPase activity | 4 | 0.016222 | 0.047273 |
| GOTERM_MF_DIRECT | GO:0003714~transcription corepressor activity | 4 | 0.016222 | 0.047481 |
| KEGG_PATHWAY | hsa04115:p53 signaling pathway | 3 | 0.012166 | 0.047638 |
