# Supplementary material for: Downregulation of miRNA-205 Expression and Biological Mechanism in Prostate Cancer Tumorigenesis and Bone Metastasis
Source: Biomed Res Int. 2020 Oct 29;2020:6037434. doi: 10.1155/2020/6037434 (PMC7646560; doi:10.1155/2020/6037434)
Supplement: Supplementary 11 — Supplemental Table S3: CDK1 expression in bone metastatic PCa samples and non–bone metastatic PCa samples. [file 6037434.f11.docx]

Supplemental Table S3. CDK1 expression in bone metastatic PCa samples and non–bone metastatic PCa samples.

| Study | Country | Year | Sample | PCa/BM | | |  | PCa/nBM | | |
| --- | --- | --- | --- | --- | --- | --- | --- | --- | --- | --- |
|  |  |  | type | N | M | SD |  | N | M | SD |
| GSE32269 | USA | 2011 | Tissue | 29 | 6.378 | 0.642 |  | 22 | 4.787 | 0.816 |
| PMID: 26000489 | USA | 2015 | Tissue | 36 | 2.879 | 0.872 |  | 2 | 1.622 | 0.667 |
| TCGA | NA | NA | Tissue | 10 | 2.176 | 0.750 |  | 20 | 1.613 | 0.963 |

Note: CDK1: cyclin-dependent kinase 1; BM: bone metastatic; M: mean; N: number; nBM: non–bone metastatic; PCa: prostate cancer; SD: standard deviation; TCGA: The Cancer Genome Atlas.
