# Supplementary material for: Downregulation of miRNA-205 Expression and Biological Mechanism in Prostate Cancer Tumorigenesis and Bone Metastasis
Source: Biomed Res Int. 2020 Oct 29;2020:6037434. doi: 10.1155/2020/6037434 (PMC7646560; doi:10.1155/2020/6037434)
Supplement: Supplementary 12 — Supplemental Table S4: Association association between miRNA-205 expression and clinicopathological parameters in PCa samples based on TCGA database. [file 6037434.f12.docx]

Supplemental Table S4. Association between miRNA-205 expression and clinicopathological parameters in PCa samples based on TCGA database.

| Clinicopathological |  | miRNA-205 expression | |  | T-test | |
| --- | --- | --- | --- | --- | --- | --- |
| parameters | N | M | SD |  | T-value | P-value |
| Group |  |  |  |  |  |  |
| Non-cancer | 52 | 9.888 | 1.674 |  | 3.370 | < 0.001 |
| Cancer | 498 | 8.719 | 2.211 |  |  |  |
| Age (years) |  |  |  |  |  |  |
| < 60 | 180 | 8.660 | 2.195 |  | −0.242 | 0.809 |
| ≥ 60 | 308 | 8.711 | 2.270 |  |  |  |
| Pathological T stage |  |  |  |  |  |  |
| T1+T2 | 187 | 9.050 | 2.129 |  | 2.796 | 0.006 |
| T3+T4 | 298 | 8.488 | 2.204 |  |  |  |
| N stage |  |  |  |  |  |  |
| N0 | 348 | 8.743 | 2.211 |  | 0.609 | 0.543 |
| N1 | 78 | 8.573 | 2.256 |  |  |  |
| M stage |  |  |  |  |  |  |
| M0 | 456 | 8.680 | 2.245 |  | 1.812 | 0.071 |
| M1 | 3 | 6.316 | 3.623 |  |  |  |
| Gleason score |  |  |  |  |  |  |
| ≤ 7 | 295 | 8.898 | 2.100 |  | 2.278 | 0.023 |
| 8 ≥ | 204 | 8.426 | 2.389 |  |  |  |
| Recurrence |  |  |  |  |  |  |
| No | 372 | 8.760 | 2.159 |  | 1.258 | 0.209 |
| Yes | 58 | 8.369 | 2.481 |  |  |  |

Note: M: mean; N: number; PCa: prostate cancer; SD: standard deviation; TCGA: The Cancer Genome Atlas.
