# Supplementary material for: Downregulation of miRNA-205 Expression and Biological Mechanism in Prostate Cancer Tumorigenesis and Bone Metastasis
Source: Biomed Res Int. 2020 Oct 29;2020:6037434. doi: 10.1155/2020/6037434 (PMC7646560; doi:10.1155/2020/6037434)
Supplement: Supplementary 13 — Supplemental Table S5: Association association between CDK1 expression and clinicopathological parameters in PCa samples based on TCGA database. [file 6037434.f13.docx]

Supplemental Table S5. Association between CDK1 expression and clinicopathological parameters in PCa samples based on TCGA database.

| Clinicopathological |  | CDK1 expression | |  | T-test | |
| --- | --- | --- | --- | --- | --- | --- |
| parameters | N | M | SD |  | T-value | P-value |
| Group |  |  |  |  |  |  |
| Non-cancer | 52 | 1.006 | 0.419 |  | −6.037 | 0.000 |
| Cancer | 498 | 1.461 | 0.754 |  |  |  |
| Age (years) |  |  |  |  |  |  |
| < 60 | 178 | 1.368 | 0.643 |  | −2.202 | 0.028 |
| ≥ 60 | 308 | 1.515 | 0.814 |  |  |  |
| Pathological T stage |  |  |  |  |  |  |
| T1+T2 | 187 | 1.177 | 0.515 |  | −7.599 | < 0.001 |
| T3+T4 | 303 | 1.630 | 0.805 |  |  |  |
| N stage |  |  |  |  |  |  |
| N0 | 346 | 1.420 | 0.698 |  | −4.064 | < 0.001 |
| N1 | 78 | 1.837 | 0.843 |  |  |  |
| M stage |  |  |  |  |  |  |
| M0 | 454 | 1.466 | 0.745 |  | −1.531 | 0.322 |
| M1 | 3 | 2.810 | 1.783 |  |  |  |
| Gleason score |  |  |  |  |  |  |
| ≤ 7 | 293 | 1.209 | 0.528 |  | −8.855 | < 0.001 |
| 8 ≥ | 204 | 1.817 | 0.876 |  |  |  |
| Recurrence |  |  |  |  |  |  |
| No | 370 | 1.399 | 0.712 |  | −4.158 | < 0.001 |
| Yes | 58 | 1.863 | 0.824 |  |  |  |

Note: CDK1: cyclin-dependent kinase 1; M: mean; N: number; PCa: prostate cancer; SD: standard deviation; TCGA: The Cancer Genome Atlas.
